# Achieving Reproducibility and Replicability of Molecular Dynamics and Monte Carlo Simulations Using the Molecular Simulation Design Framework (MoSDeF)

**DOI:** 10.1021/acs.jced.5c00010

**Published:** 2025-05-27

**Authors:** Nicholas C. Craven, Ramanish Singh, Co D. Quach, Justin B. Gilmer, Brad Crawford, Eliseo Marin-Rimoldi, Ryan Smith, Ryan DeFever, Maxim S. Dyukov, Jenny W. Fothergill, Chris Jones, Timothy C. Moore, Brandon L. Butler, Joshua A. Anderson, Christopher R. Iacovella, Eric Jankowski, Edward J. Maginn, Jeffrey J. Potoff, Sharon C. Glotzer, Peter T. Cummings, Clare McCabe, J. Ilja Siepmann

**Affiliations:** 1 Interdisciplinary Materials Science Program, 5718 Vanderbilt University, Nashville, Tennessee 37235-0106, United States; 2 Department of Chemical Engineering and Materials Science, 172299 University of Minnesota, Minneapolis, Minnesota 55455-0132, United States; 3 Department of Chemistry and Chemical Theory Center, 172299 University of Minnesota, Minneapolis, Minnesota 55455-0431, United States; 4 Department of Chemical and Biomolecular Engineering, 5718 Vanderbilt University, Nashville, Tennessee 37235-1604, United States; 5 Department of Chemical Engineering, 2954 Wayne State University, Detroit, Michigan 48202-4050, United States; 6 Atomfold, Smithfield, Pennsylvania 15478, United States; 7 Department of Chemical and Biomolecular Engineering, 6111 University of Notre Dame, Notre Dame, Indiana 46556, United States; 8 Micron School of Materials Science and Engineering, Boise State University, Boise, Idaho 83725, United States; 9 Department of Chemical Engineering, 1259 University of Michigan, Ann Arbor, Michigan 48109-2800, United States; 10 Biointerfaces Institute, 1259 University of Michigan, Ann Arbor, Michigan 48109-2800, United States; 11 School of Engineering and Physical Sciences, 3120 Heriot-Watt University, Scotland EH14 4AS, United Kingdom

## Abstract

Molecular simulations are increasingly used to predict thermophysical properties and explore molecular-level phenomena beyond modern imaging techniques. To make these tools accessible to nonexperts, several open-source molecular dynamics (MD) and Monte Carlo (MC) codes have been developed. However, using these tools is challenging, and concerns about the validity and reproducibility of the simulation data persist. In 2017, Schappals et al. reported a benchmarking study involving several research groups independently performing MD and MC simulations using different software to predict densities of alkanes using common molecular mechanics force fields [ J. Chem. Theory Comput. 2017, 4270−4280 28738147 10.1021/acs.jctc.7b00489]. Although the predicted densities were reasonably close (mostly within 1%), the data often fell outside of the combined statistical uncertainties of the different simulations. Schappals et al. concluded that there are unavoidable errors inherent to molecular simulations once a certain degree of complexity of the system is reached. The Molecular Simulation Design Framework (MoSDeF) is a workflow package designed to achieve TRUE (Transparent, Reproducible, Usable-by-others, and Extensible) simulation studies by standardizing the implementation of molecular models for various simulation engines. This work demonstrates that using MoSDeF to initialize a simulation workflow results in consistent predictions of system density, even while increasing model complexity.

## Graphical abstract

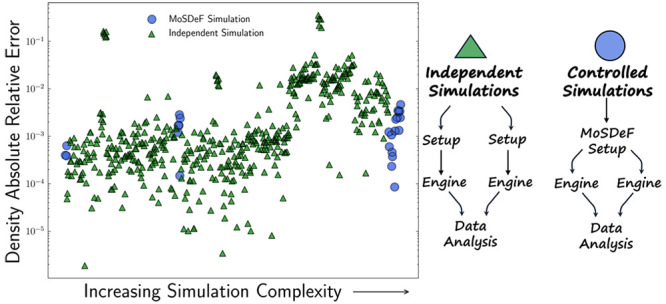

## 1 Introduction

Molecular simulation (MS) has become an indispensable tool across many disciplines to investigate and predict the thermodynamic, transport, and structural properties of chemical, material, and biological systems.^1–3^ Over the past several decades, MS has progressed, serving in various roles, such as computer experiments to test analytical theories,^4^ analysis of trajectories to complement experimental measurements,^5^ a source of reliable physical properties data,^6^ and exploratory work for the design of novel materials.^7–9^ The continued increases in computing power have precipitated development of numerous community-available simulation engines, force fields (or potential energy functions), and analysis tools.^10–28^ The improvements in accessibility of these methods have resulted in an explosion of MS research due to the substantial reduction in the barrier to entry.

The rise of well documented, open-source, and community-developed simulation codes has undoubtedly been a driver for much of the growth but has also resulted in several issues. As with many other arenas of knowledge, the expansion of the MS community has reduced the average knowledge of the aggregate users, whereas only a few experts who distribute the complex source codes are able to distill the algorithms down into descriptions of their impact on the simulation methodology. Concurrently, the familiarity of these codes has allowed researchers to simply identify the code and force field implementation by name in their work and presume that the audience has the domain knowledge to parse this documentation and understand the calculations that were performed. These conflicting expectations have contributed to an effect referred to as the “reproducibility crisis”, where incomplete information leads to challenges for researchers attempting to reproduce the results in published work.^29^ We note that this reproducibility crisis is not unique to computational based research and is a burgeoning topic of conversation in the academic community as a whole.^30–32^

Several works have attempted to address and document the state of the current MS reproducibility crisis. Sometimes seemingly benign details of the simulation protocol can have large effects, particularly when the stability of phases is compared with small free energy differences. For example, the disagreements between publications regarding the presence of a second liquid–liquid coexistence region for supercooled water was recently prominent in the literature and resolved following collaboration to share simulation codes and input files.^33^ Other work has been more broad, such as the 2016 paper from Wong-Ekkabut and Karttunen discussing the relevancy of different user errors in the MS processes for soft matter, including examples of errors in thermostatting, the importance of choosing proper Lennard-Jones (LJ) cutoffs, and the differences between long-range electrostatic calculation methods.^34^ Collective efforts reported by Schappals et al.,^35^ also referred to herein as the round robin (RR) study, approached the topic through the use of a central agency, which defined a simulation “task” that was sent to different research groups using different simulation codes and then asked the groups to report their results without intercommunication. What is notable about the RR study is the effort to allow groups to independently set up their MS simulations with only the information on the force fields available in the original publications and their knowledge of the selected MS engines, and therefore, this enabled a look into the scale of the possible errors introduced with current MS practices. The study showed that most groups were able to obtain data in agreement within a “practical” viewpoint (i.e., deviations between different MS predictions at a level smaller than typical deviations from experimental data) but far outside of the statistical errors of the individual simulations.

While much work has gone into identifying and quantifying these errors,^36^ equally important are attempts to prevent them. Work from the authors herein have highlighted the importance of four principles for a MS workflow: the workflow should be transparent, reproducible, usable by others, and extensible (TRUE).^37^ These four principles, adopted from generalized best practices in already well established computational research fields, are proposed standards to enable a well-defined approach for publishing research in a consistent manner such that the community can easily access the exact methods explored in the work. The result of this is the MoSDeF (**Mo**lecular **S**imulation **De**sign **F**ramework) toolkit, which is a collection of open-source, integrated python packages that standardizes the initialization of MS studies across simulation engines.^38–42^

The MoSDeF toolkit was conceived to support some of the widely used open-source simulation engines. The goal was to allow users to initialize the topology of the system, define the thermodynamic constraints (i.e., the statistical–mechanical ensemble and the state point), and apply a force field to the specified system in a standardized manner while switching with ease between different MS engines. The scriptability of the software allows users to define a set of inputs and complete the entire simulation process using workflow management tools such as the signac framework.^43,44^ The defined nature of this procedure incorporates key ways to avoid potential errors of a MS workflow: (1) inputs are defined in a singular place and are able to be referenced at any point in the workflow as project metadata, (2) the same system topology can be output to a variety of simulation engines through conversions and is handled by thoroughly tested open-source software, and (3) the force field parameters and generalized simulation files are stored in accessible places. Thus, the actual implementation of the MS model used is well documented and can be shared and run across different computational platforms. In order to evaluate the effect of these principles, the following question is raised: To what extent does the use of MoSDeF enable more reproducible MS studies? In this work, we start with the same simple question asked by Schappals et al.:^35^ “whether they [different MS engines] agree within the statistical uncertainty of the individual data.” In order to scrutinize the benefits of the TRUE principles,^37^ six MS engines supported by MoSDeF, divided equally between molecular dynamics (MD) and Monte Carlo (MC), were chosen and five chemical compounds represented by models with increasing complexity were simulated in the isothermal–isobaric (*NpT*) ensemble. By comparing the relative error in the results of molecular simulations prepared using the MoSDeF toolkit, we demonstrate how using a standardized MS workflow, starting from system initialization to data analysis, can help achieve an acceptable degree of replicability.

In an ideal world, where all available MS engines have correctly and consistently implemented models and are employed without bugs, when given an exact set of simulation input and atomic coordinates (and velocities), one would expect to obtain the exact same system energy (which could be further decomposed for pairwise-additive molecular mechanics force fields) and atomic forces down to the machine precision level. In a simulation, a user is trying to measure a property (*x* ^sim^) by applying a force field, a set of thermodynamic constraints, and a MS engine to sample the trajectory of the system and obtain the true value of this property for a given model (*x* ^tm^). Unfortunately, multiple sources of errors are known to exist during practical model execution,^45^ which may cause noticeable differences between *x* ^sim^ and *x* ^tm^. These are generally categorized in three ways: machine-precision (a.k.a. rounding) and, for MD simulations, finite-difference (a.k.a. time step) errors leading eventually to diverging trajectories (and, in severe cases, systematic deviations from the true trajectory), completeness errors caused by incomplete sampling of the statistical–mechanical phase space (e.g., simulations do not reach relevant equilibrium states), and execution errors, which can have many different sources. Schappals et al. provide a breakdown of some of the errors relevant to MS.^35^ It should be emphasized here that we distinguish between errors that lead to answers significantly different from *x* ^tm^ and statistical errors that lead to imprecise results but that should still encompass *x* ^tm^ within their (statistical) error bounds.

However, many in the MS community lack an understanding of the acceptable tolerances for these different types of errors, including what methodological errors in the MS process provide an approximation of *x* ^tm^ that can be considered an acceptable solution. It should be noted that the current work is not concerned with the inaccuracy of the model which arises from the force field not capturing the true many-body interactions or the sampling not accounting for nuclear quantum effects. The expected outcomes of carrying out MS for a specified system can be broken down into three categories. **Repeatability** is expected when the same observer is using the same MS engine (software version, hardware, and compiler), force field, and system specification but initializing the MS from a different microstate (set of atomic positions and velocities) or random number seed for MC, which should result in predictions differing solely within the statistical uncertainty from the ensemble trajectories that are being sampled. **Reproducibility** should be found when using the same MS engine (i.e., same source code) and input files, but different observers, hardware, and/or compiler are used to perform the sampling, which may result in additional machine-precision and compiler errors (this is a challenge that requires better software engineering). Trajectories in simulations (whether MD or MC) will diverge according to Lyapunov theory over the course of the simulation even when the same simulation code is used on the same computer with the exact same initial conditions due to rounding error in computer calculations (which can be controlled, at considerable computational cost, by using IEEE-compliant arithmetic); the problem is exacerbated on parallel or multicore computers as arithmetic operations are performed in unpredictable order owing to random differences in message-passing times. **Replicability** arises when performing MS on the same model system, with the same thermodynamic constraints, and the same force field (as described in a publication and its Supporting Information), but using a different MS engine, hardware, compiler, and input files suitable for the different engine. Whereas achieving repeatable and reproducible simulations is fairly straightforward, performing replicable MS is very much a challenge, and the field lacks well-formulated expectations and needs guidance on the achievable limits. For all intents and purposes, the acceptable error will be referred to as **practical replicability** in this work. Plainly, statistical uncertainties are acceptable and cannot be avoided given a finite amount of computer time but can be handled with ubiquitous statistical techniques. Gross user errors or significant software bugs are unacceptable, as these result in a misrepresentation of the model system or failure to properly sample the thermodynamic space. The gray area resides with disparities introduced by software and algorithm implementations, which are typically motivated by a desire to reduce the computational cost for computing *x* ^sim^ (e.g., number of bits used to store positions and truncation of intermolecular potentials). This trade-off is predicated on the hope that the systematic errors introduced might be smaller than or on the order of the unavoidable statistical uncertainties for the simulation and of the inaccuracy of the model (i.e., in most cases, the MS community aims to predict the properties of real chemical systems and not specific models). Because the error sources are coupled together, evaluating the software and implementation errors is difficult. Schappals et al. reached the conclusion that the inherent errors in MS make it challenging to obtain practically replicable results from two different simulation engines and/or research groups for the same model system.^35^ Specifically, their conclusions state: “The collected data demonstrate that systematic errors are important in molecular simulations. We emphasize that it must be the goal to entirely avoid systematic errors in molecular simulations. However, there should be no doubt that fully achieving this goal is practically impossible.”^35^ In this study, we explore whether the use of MoSDeF by disparate groups using multiple MS codes can result in practical replicability.

To this end, in this work, we show that practical replicability in MS predictions is an achievable goal utilizing the MoSDeF toolkit, which eliminates some of the typical sources of discrepancies between MS simulations, such as differences in force field parameters. We address safety checks that can be undertaken and workflows that can be designed to surmount the challenges discussed in the RR study. In particular, we show that software bugs and algorithm implementations in widely used software packages should not be the main source limiting practical replicability. On the other hand, systematic errors can be caused by imperfect model implementation (e.g., differences in physical constants used for unit conversions). This work led to addressing some of the sources of systematic errors.

Following the lead of Schappals et al.,^35^ this study focuses on the prediction of the specific density for a given system using simulations in the isobaric–isothermal (*NpT*) ensemble. We examine five prototypical compounds (see Table 1) that range from a single-site methane model (i.e., no internal degrees of freedom) to rigid water and benzene models (posing different challenges to maintaining their rigid structures) and flexible *n*-pentane and ethanol models. Three MD (LAMMPS,^10^ GROMACS,^11,12^ and HOOMD-blue^13^) and three MC (CASSANDRA,^14,15^ GOMC,^16,17^ and MCCCS-MN^18,19^ ) engines are used to sample the trajectories. To facilitate future studies, complete documentation is provided to allow users to regenerate the data and extend the simulations. Instructions and scripts to replicate the simulations and analyses are provided in Section 1 of the Supporting Information.

**Table 1:** Chemical Compounds, Models, and Purity^a^

chemical name	chemical or linear formula	CAS number	model
methane	CH_4_	74-82-8	TraPPE-UA^46^
*n*-pentane	CH_3_(CH_2_)_3_CH_3_	109-66-0	TraPPE-UA^46^
benzene	C_6_H_6_	71-43-2	TraPPE-UA^47^
water	H_2_O	7732-18-5	SPC/E^48^
ethanol	C_2_H_5_OH	64-17-5	OPLS-AA^49^

a All chemical samples are pure as specified by their respective input files.

## 2 Methods

When performing a MS study with interactions described at the level of molecular mechanics (instead of being obtained from an on-the-fly electronic structure calculation), there are three components that need to be properly specified: system, molecular model including force field parameters, and algorithm and trajectory parameters. The specification of the system in MS studies is closely analogous to that in experimental studies. Foremost, one has to specify the composition of the system: chemical compounds including, if applicable, the crystal form of a compound (e.g., the specific structure of a zeolite) or the grafting density for a ligand-modified surface, the mole fraction for each chemical compound (or its chemical potential), and the thermodynamic constraints for the chosen statistical–mechanical ensemble. The current study focuses on the prediction of the liquid-phase density for unary (i.e., single-component) systems, which can be achieved by simulations in the isobaric–isothermal ensemble where the total number of molecules (in addition to the mole fractions, see above), the applied (external) pressure, and the absolute temperature constitute the thermodynamic constraints.

The molecular model for a given chemical compound specifies the number of interaction sites (which can be larger than, equal to, or smaller than the number of atoms), their connectivity (i.e., the geometry of the molecular model), and the vibrational degrees of freedom allowed to be sampled in the MS trajectory. For example, there are popular models for water that utilize 5, 4, 3, and 1 interaction site per molecule or that group 4 water molecules into a single interaction site.^48,50–54^ Reducing the number of interaction sites per molecule increases the computational efficiency. Similarly, treating some of the connectivity as rigid (e.g., fixing the distance or “bond” length between two interaction sites) increases the computational efficiency. The force field is a set of equations that describe intra- and intermolecular interactions. Molecular mechanics force fields utilize a small set of equations (e.g., a Lennard-Jones potential to describe the “non-bonded” repulsion and dispersion interactions between sites on different molecules or separated by four or more bonds within a molecule). The parameters for the equations can be derived by fitting them to experimental data, quantum mechanical data, or their combination. Since experimental data are usually only available for macroscopic properties measured at a given state point, the “empirical” force fields are implicitly attempting to match free energies of the target system and usually employ relatively few parameters. In contrast, quantum mechanics can yield forces for each nucleus in each configuration and, hence, allows for force fields (usually treating each atom as a separate entity, i.e., not defining molecules) with a great number of parameters that may be incorporated into a complex machine-learned model (e.g., a deep neural network). For compounds that consist of multiple types of interaction sites and for mixtures, the force field also includes combining rules for how parameters for interactions between unlike beads can be determined from those for like beads. Importantly, the combining rules afford force fields some degree of transferability where new compounds can be built from a set of sites (e.g., methyl and methylene groups to build linear alkanes).^46,49^ Furthermore, the force field includes information on truncation of interactions at larger distances (and, sometimes, also offsets to avoid discontinuities at the truncation distances) and how interactions beyond the truncation cutoff are accounted for. Since the force field is parametrized to reproduce the available data using a given set of specifications, the only “correct” way to use the force field is to exactly follow the defined specifications; any changes to these specifications, no matter the scale of their effects, can be thought of as a modified form of the original force field. Although changing the specifications can be thought of as being akin to introducing impurities, sometimes the adjusted specifications might lead to an increase in accuracy (i.e., better agreement with experimental data). Another often overlooked problem when aiming for replicability of the specific density with small tolerance is that the masses for the atoms and pseudoatoms need to be reported (whereas these are not needed when reporting the number density for classical simulations).

The implementation is the final step of the process and allows for some flexibility in the methodology. Simply put, practical simulations require trade-offs between accuracy, precision, and accessibility, which are evaluated based on circumstance, heuristics, and experience. A specific simulation engine, which employs either MD or MC to propagate trajectories, has predefined options for many of these choices, which are some subsets of all the published simulation methods. Users must make judgments to compromise these trade-offs, where inherent error is built into the procedures and “reasonable” decisions must be made. These decisions include simulation specifications such as the MD time step and thermostat/barostat sampling algorithms, the MC move types and probabilities, and the methods used to evaluate induction interactions (e.g., polarization in the form of Drude oscillators) and the long-range contributions for first-order electrostatic interactions (Coulomb interactions of fixed charges, dipoles, and higher terms) and dispersion interactions. These parts of the simulation process are defined below, and in this work, differences in some of these simulation inputs are explored to understand the relative scales of their effects on the resultant densities.

### 2.1 Molecular Models and State Points

We investigate methane, *n*-pentane, benzene, water, and ethanol representing a sequence of molecular models of increasing complexity (see Figure 1). For each molecule, the choice of molecular representation, which is either all-atom (AA, each atom treated as distinct interaction site) or united-atom (UA, nonpolar hydrogen atoms merged with their bonded heavy atom into a pseudoatom), the bonded components (defining specific intramolecular interactions), and the state of the system constitute the *model*. Methane, *n*-pentane, and benzene represented at the UA level with parameters taken from the TraPPE-UA force field are simulated at one temperature and elevated pressure, while AA models for water and ethanol with interactions described by the SPC/E and OPLS-AA force fields, respectively, are simulated at three temperatures and atmospheric pressure.

**Figure 1:**
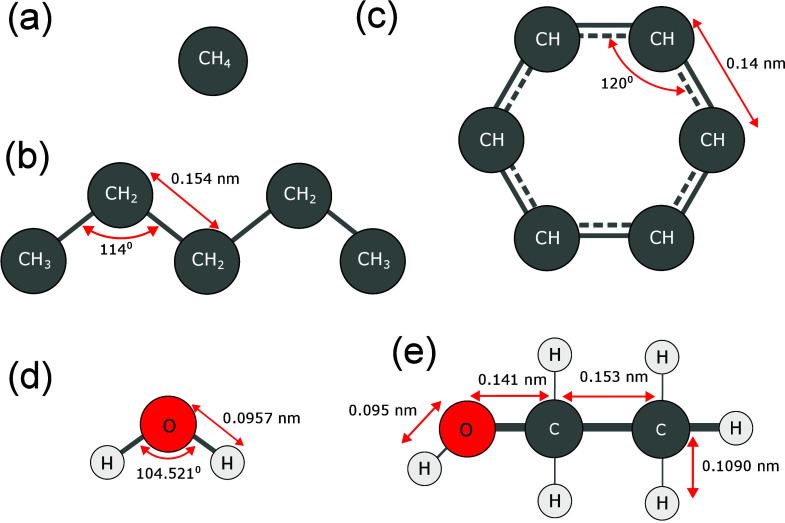
Structures of the five models with each atom type labeled: (a) TraPPE-UA methane, (b) TraPPE-UA *n*-pentane, (c) TraPPE-UA benzene, (d) SPC/E water, and (e) OPLS-AA ethanol.

The state points for the simulations are reported in Table 2. Simulation conditions for methane, *n*-pentane, and benzene were selected to ensure that the systems are in the liquid phase (at least when cutoff and long-range conditions match those used during the force field parametrization). Each system’s temperature was selected to fall significantly below the corresponding critical temperature, yet not to be excessively low so as to impede configurational space sampling. Thus, simulation temperatures, *T*, were chosen such that the reduced temperature, *T*_r_ = *T*/*T*_crit_, falls in the range from 0.7 to 0.8, with the specific value determined by the available literature data for the saturated vapor pressure, *p*_vap_. For *n*-pentane and benzene, the pressure was set to twice the value of *p*_vap_ reported previously.^46,47^ For methane, two-box Gibbs ensemble Monte Carlo simulations were conducted at *T* = 140 K using GOMC, and again *p* was set to twice *p*_sat_. As for water and ethanol, simulations were conducted at atmospheric pressure and three temperatures encompassing ambient temperature with a spread of 20 K in either direction.

**Table 2:** Description of the Systems Studied^a^

system	*N*	*T* [K]	*p* [kPa]	FF name	*r*_cut_ [Å]
methane–TraPPE	900	140	1318	TraPPE-UA^46^	14
pentane–TraPPE	300	372	1402	TraPPE-UA^46^	14
benzene–TraPPE	400	450	2260	TraPPE-UA^47^	14
water–SPC/E	1100	280, 300, 320	101.325	SPC/E^48^	9
ethanol–OPLS	500	280, 300, 320	101.325	OPLS-AA^49^	10

a Number of molecules, *N*; system temperature, *T*; external pressure, *p*; force field name; and pairwise interaction cutoff distance, *r*_cut_ are provided. The molecular weights of each of the interaction sites are given in Table S1.

### 2.2 Force Fields

The hydrocarbon models considered in this work are taken from the united-atom version of the Transferable Potentials for Phase Equilibria (TraPPE-UA) force field^46,47^ parametrized using experimental vapor–liquid coexistence data. The extended simple point charge (SPC/E) model^48^ is used to represent water, and the all-atom version of the Optimized Potentials for Liquid Simulations (OPLS-AA) model^49^ is used for ethanol. These models offer a set with increasing complexity to allow for a range of possible engine-by-engine differences to appear. TraPPE-UA methane is the simplest model, as the five-atom molecule is reduced to a single LJ interaction site with no rotational or vibrational degrees of freedom. The five-site TraPPE-UA pentane model adds harmonic bond bending and cosine series dihedral and intramolecular 1–5 LJ potentials, while the bond length is fixed for MC engines and constrained for MD engines. The six-site TraPPE-UA benzene and three-site SPC/E water models both use rigid geometries that pose additional challenges beyond a simple bond length constraint. Here it should be noted that we did not test the nine-site TraPPE-UA model (with out-of-plane partial charges)^47^ because some of the MD engines cannot handle nonplanar rigid bodies. The SPC/E water model also adds partial charges interacting with a Coulomb potential. Finally, the nine-site OPLS-AA ethanol model provides a branched architecture and is the most complex molecule studied here, which includes bond stretching, angle bending, dihedral rotation, and Lennard-Jones and Coulomb interactions. For this model, fixed bond lengths were used for all three MC engines, and the effects of that choice were then compared for LAMMPS and MCCCS-MN, which can simulate this model using either fixed bond lengths/bond constraints or harmonic bond stretching potentials. To ensure correct bond lengths and angles for rigid and constrained models, the constrainmol utility was used.^55^ The exact force field parameters used in the study are made available through the GitHub repository for this project.^56^

### 2.3 Implementation Details

The six simulation engines considered are the MC codes CASSANDRA,^14,15^ GOMC,^16,17^ and MCCCS-MN,^18,19^ and the MD codes HOOMD-blue,^13^ LAMMPS,^10^ and GROMACS.^11,12^ The first four of these simulation engines are developed and maintained by groups collaborating in the MoSDeF project, thus providing the additional flexibility of code modifications and debugging. The use of a diverse array of engines allows us to explicitly evaluate the consistency of the predicted properties and whether differences in methodological implementations lead to marked differences in output, even when MoSDeF workflows are used to generate the input and parameter files. Table S2 provides the native units for each of the six energies necessary for this interconversion.

#### 2.3.1 General Molecular Dynamics Details

Molecular Dynamics (MD) simulations are performed with three different engines: LAMMPS, GROMACS, and HOOMD-blue which all run on a computing cluster located at Vanderbilt University. Simulations are performed in the *NpT* ensemble to calculate single-phase properties for the five models. In all cases, the physical validation package^57^ is used to check that the kinetic energy distribution obeys the ensemble statistics (see Section S3 and Figures S1–S3 of the Supporting Information). Although the simulations use the same molecular models (force field parameters including cutoff distance and, if applicable, long-range corrections), there are some differences in the methodological implementations (smoothing of potentials near the cutoff, force from long-range correction on box length, Ewald summation, thermostat and barostat implementations, time step, and equilibration procedure). These variations are unavoidable since each engine was developed and optimized for certain types of simulations and, hence, necessitate different implementations. The next section contains an elaboration of these differences.

#### 2.3.2 LAMMPS Specific Details

The LJ and Coulomb energies are calculated using the force field specified cutoff distance. The particle–particle–particle mesh (PPPM) solver^58,59^ with order 5 and a relative tolerance in force of 1 × 10^–5^ is used for Coulomb interactions present for the water and ethanol systems. If included for a given force field, then the analytical tail corrections are used for the LJ potential. For SPC/E water, the SHAKE algorithm with a tolerance of 1 × 10^–5^ is used to constrain the bond length and bending angle.^60^ Benzene–TraPPE is constrained using rigid-body integration. LAMMPS is unable to perform chained bond constraints for oligomers, so simulations for the TraPPE pentane model are not carried out. LAMMPS simulations are initialized using 3 cycles of the conjugate gradient algorithm and an *NVE* time integration with a maximum displacement of 0.1 Å. A time step of 2 fs is used for all systems except ethanol, for which a 1 fs time step is used to account for the faster motion of hydrogen atoms. Then *NVT* and *NpT* integration are used to equilibrate the system. These simulations use a Nosé-Hoover thermostat^61^ with a characteristic damping time of 100 time steps. For the *NpT* stage, a three-chained Nosé-Hoover barostat^62^ is used following the Martyna–Tuckerman–Klein (MTK) method chaining 3 barostats to control oscillations in the pressure.^63^ A characteristic damping time of 1000 time steps is used for the barostats. Equilibration is assessed using pymbar^64–66^ to find decorrelated data. At least 100 configurations or 80% of data during a 2 ns equilibration simulation is required to be equilibrated. If this requirement is met, the trajectory is transitioned to an *NpT* production stage. The production stage is run for 10 ns, and simulation data are collected every 10 ps, which is then used for density sampling as described in Section 2.4. LAMMPS version 23 June 2022 was used for all simulations.

#### 2.3.3 GROMACS Specific Details

For applicable systems, i.e., water–SPC/E and ethanol–OPLS, the PPPM algorithm was used to calculate electrostatic potential energy with the Fourier spacing set to 0.1 nm. The GROMACS simulation workflow is started with an energy minimization step, followed by a pre-equilibration in the *NVT* ensemble with a duration of 5 ns. Thereafter, an equilibration in the *NpT* ensemble for at least 1 ns, followed by the production stage with a duration of 5 ns. pymbar^64–66^ (version 3.0.5) is used to assess equilibration requiring here at least 100 uncorrelated data points or 80% of the equilibrated data. For both the *NVT* and the *NpT* ensemble, the Nosé-Hoover thermostat is utilized to maintain the temperature at the target value. For the *NpT* ensemble, the Parrinello–Rahman barostat is used to maintain the pressure.^67^ The energy minimization is done using the steepest descent algorithm, while a leap-frog integrator is used for all other simulation steps. The LINCS constraint algorithm is used for the four bonds of the TraPPE pentane model.^68^ The *lincs*–*order*, which defines the number of matrices used in the expansion of the constraint forces, is set at 4. The SHAKE constraint algorithm is used to constrain all bond lengths and the bending angle in the simulations for the SPC/E water model. The SHAKE relative tolerance is set at 1 × 10^–5^. We note that SHAKE constraints can not be used with the steepest descent algorithm for energy minimization. Hence, for the simulations of SPC/E water, the energy minimization step is replaced with an *NVT* simulation of 5 ns. A time step of 1 fs is used for all systems, and the coordinates are written to file every 10000 time steps while the energy is written out every 1000 time steps during the production *NpT* run. Single precision GROMACS 2020.6 is used.

#### 2.3.4 HOOMD-blue Specific Details

The long-range electrostatics are calculated via the PPPM method with order 5 and stencil grids of 32 × 32 × 32 for water–SPC/E and 24 × 24 × 24 for ethanol–OPLS.^58,69^ Independent random initial configurations are generated by first packing a large volume (twice the target starting volume box lengths) with uniformly distributed molecule positions, followed by a 1 ns nonequilibrium simulation at the set-point temperature during which the box axes are scaled down to the target starting volume. From these pre-equilibrated conditions, equilibrium *NpT* simulations are performed using the Bussi^70^ thermostat for 2 × 10^7^ time steps while sampling every 1000 time steps. These are performed in batches until a production trajectory with 80% equilibration and 100 independent samples is obtained. All simulations are run with a 1 fs time step with HOOMD-blue base units of kJ/mol, nm, and amu. The rigid molecules, benzene–TraPPE and water–SPC/E, are integrated via rigid body dynamics.^71^ Double precision HOOMD-blue v4.0 is used. We note that as the long-range correction simulations in Section 3.7 were run before HOOMD-blue v4.0 was released, those simulations are run with the MTK thermostat^62^ in v3.11.

#### 2.3.5 General Monte Carlo Details

Monte Carlo simulations in the *NpT* ensemble are conducted with three different simulation engines, Cassandra, GOMC, and MCCCS-MN, for the molecules and force fields mentioned in Table 2. Cassandra is run on the University of Notre Dame cluster, GOMC is run on the Wayne State University cluster, and MCCCS-MN is run at the Minnesota Supercomputing Institute. To reduce the number of runtime parameters that control the sampling efficiency for MC simulations, a set of common (but not necessarily efficient) move probabilities is used for the MC simulations. That is, volume moves are selected with a probability of either 0.01 or 2.5/*N* with the remainder of the moves distributed equally over molecular translations (all molecules), rigid-body rotations (all molecules except methane), and regrowth (only *n*-pentane and ethanol). By default, all molecules are simulated with fixed bond lengths, not using atom displacement moves or bond length sampling during the regrowth of semiflexible molecules. The effect of keeping the bond lengths fixed on the system density was studied in detail for OPLS-AA ethanol (Section 3.6), a model that has flexible bonds but is often simulated with fixed bond lengths in MC engines. All MC engines employ analytical tail corrections to energy and pressure for the Lennard-Jones interactions. The Ewald sum method^72,73^ is used to compute Coulomb interactions. The number of reciprocal space vectors (*k*-vectors) is consistent between engines to achieve a summation accuracy of 1 × 10^–10^. All simulations utilize a hard inner cutoff of *r*_min_ = 1.0 Å, i.e., trial moves that place atomic nuclei within *r*_min_ are automatically rejected.

The MC workflows involve a series of simulations to reach equilibrium. The length of the MC simulations is given in MC cycles (MCCs), where a cycle consists of *N*, the number of molecules randomly selected moves. The first and second stages are carried out in the *NVT* ensemble. The first stage consisting of 5000 MCCs utilizes a high temperature of 1000 K to ensure that a disordered state is reached from the initial configuration that has molecules placed on lattice positions and, depending on the engine and molecule, with common or random orientations. The second state, also consisting of 5000 MCCs, brings the system to the target temperature. The third stage involves simulations in the *NpT* ensemble to allow the system to reach equilibrium at the target temperature and pressure. The equilibration simulations are each run for 40000 MCCs, and pymbar is used to test whether equilibrium is reached for at least the last 10000 MCCs. If not, then up to two additional equilibration periods of 40000 MCCs are used. In the fourth stage, the final production simulation is conducted for 120000 MCCs at the target temperature and pressure. Simulation data and snapshots are collected every 10 MCCs. There are subtle differences among the simulations conducted by the three MC engines, which are described below.

#### 2.3.6 Cassandra Specific Details

Cassandra utilizes a fragment-based configuration-biased method to sample the internal degrees of freedom of molecules. Details of this method can be found elsewhere.^14,15^ For flexible molecules (pentane–TraPPE and ethanol–OPLS), a library of 100000 fragment conformations are pregenerated utilizing atom displacement moves. The atom displacement algorithm generates a bond angle distribution while preserving fixed bond lengths.^15^ During regrowth moves, 50 dihedral trials are used.

#### 2.3.7 GOMC Specific Details

For flexible molecules (pentane and ethanol), GOMC uses the coupled–decoupled configurational-bias method for the regrowth moves.^74^ Twelve trial positions are used for the first bead, and 10 trial positions are used for each remaining bead in the molecule. 50 trial bond and dihedral angles are used.

#### 2.3.8 MCCCS-MN Specific Details

During the equilibration stages, MCCCS-MN automatically adjusts the maximum displacements for volume, translation, and rotation moves to yield acceptance rates matching a target value (here, set to 40%). It should be noted that MCCCS-MN uses only a single Cartesian coordinate/axis for the translation/rotation moves, but the maximum displacements are the same irrespective of the direction for isotropic systems. For the SPC/E water and OPLS-AA ethanol simulations, the volume move probability is set to 2.5/*N*, where *N* is the number of molecules, to achieve one accepted volume move per MCC. For flexible molecules (pentane and ethanol), MCCCS-MN uses the coupled–decoupled configurational-bias algorithm with dual-cutoffs for the regrowth moves.^74,75^ Sixteen trial positions are used for the first bead, and 8 trial positions are used for each subsequent step in the regrowth. 100 torsional angles are considered during the regrowth. The Coulomb energy for water and ethanol is calculated by utilizing the Ewald summation technique.^72,73^ The screening parameter, κ is set as 3.6/*r*_cut_ to achieve a relative accuracy of 1 × 10^–5^, where *r*_cut_ is the cutoff distance used for both LJ and Coulomb interactions, and the number of reciprocal lattice vectors is set to int­(κ*L*_box_) + 1, where *L*_box_ is the box length. For simulations in the *NpT* ensemble, the number of reciprocal vectors can change in conjunction with the box length.

It is important to note that MCCCS-MN is not yet integrated with Foyer. Thus, the force field parameters in MCCCS-MN have to be manually input by the user, in contrast to the other simulation engines in this study, which leverage Foyer to load the force fields. Other MoSDeF/signac operations (initial structure generation, simulation conduction, and analysis) for MCCCS-MN follow those used for the other engines. For reporting densities, MCCCS-MN calculates the specific density at each time step using double precision variables while giving configurations the same weight.

### 2.4 Density Sampling and Data Analysis

For the MD simulations, once pymbar determines that equilibration is reached, the entire production trajectory is used for trajectory averages. For the MC simulations, the importance sampling algorithms require that every configuration contributes equally to the ensemble average. Here, we follow the approaches built in natively into the different engines. For example, MCCCS-MN calculates the number and specific densities after every move (irrespective of whether the move is accepted or rejected) and adds these in double precision to their respective accumulators. It should be noted that this on-the-fly is a trivially inexpensive operation compared to the calculation of the energy differences between configurations.

For each engine, system, and state point, the mean density values obtained for each of the 16 independent replicas are averaged to generate a sample mean and a 95% confidence interval (CI), which is compared to test for statistical similarity across the different engines. Values are also compared via their relative percentage deviation, δ, calculated with respect to the unweighted average density, ρ̅_all_, from all the simulation engines:

$$\delta =100\times \left({\mathrm{\rho ̅}}_{\mathrm{engine}}-{\mathrm{\rho ̅}}_{\mathrm{all}}\right)/{\mathrm{\rho ̅}}_{\mathrm{all}}$$ 1

This procedure allows us to estimate the extent of implementation differences for the simulation engines considered in this work.

## 3 Results

### 3.1 Single Point Energy Calculations

As discussed in the introduction, there are numerous sources of errors and ways to classify them. The complexity of errors relevant to MS methods has led to the sense that these errors may be undiagnosable and a degree of resignation or dismissal of their impact on MS studies.^35,76,77^ However, we propose that a carefully controlled and documented approach leveraging MoSDeF allows us to reduce possible errors and pinpoint, and then mitigate, the sources of some of the errors.

In the simulation pipeline, MoSDeF defines and controls numerous simulation inputs, including the initial configuration and model parameters. To assess whether these inputs have been defined in equivalent fashions for each simulation engine, we started by an examination of single-point energies for the five systems studied in this work. The single-point energy calculations remove finite time step (i.e., integration) and sampling errors from consideration. Due to the simplicity of the single-point energy calculations and output data, agreement in single-point energies assures that we may discount initialization steps and force field implementation from the major sources of errors.

To ensure that all engines use the same configuration for the single-point energy calculation, a library of system configurations containing the same number of molecules as the subsequent simulations (see Table 2) is created using PACKMOL to place molecules randomly in a periodic box. Although the box sizes are determined using the densities for these systems from the literature, the configurations do not represent equilibrated states. The snapshots are stored in JSON format, which can be loaded in mBuild without any loss of information and can be exported to the formats expected by the different simulation engines.

For rigid or semiflexible molecules, it is essential to check whether PACKMOL generates valid configurations, and the initial configurations are reported in Table S3. We find agreement with the force field specified length to a relative error of 1 × 10^–7^. This is impactful because some engines may not recompute bond lengths for bonds to be known of fixed length, thus any deviation from the correct bond length will cause erroneous calculations of bending and dihedral angles because unit vectors are not found when scaling the bond vector by the fixed bond length.

The results for single-point energy calculations for the five systems are summarized in Table 3 (energy decompositions are provided in Tables S4–S8). Absolute system energies agree to 5 significant digits, and the relative deviations agree to 0.004% for all systems. For the molecules without partial charges, four of the engines yield single-point energies that deviate by less than 2 × 10^–4^%.

**Table 3:** Single Point Energy for Identical Initialized Snapshots, Compared As Relative Error ((*U* – *U̅*)/*U̅*) of the Total Potential Energy Across the 6 Engine Potential Energies (×10^5^) for Each Molecule

Error× 10^5^					
simulation engine	methane–TraPPE	pentane–TraPPE	benzene–TraPPE	water–SPC/E	ethanol–OPLS
LAMMPS (MD)	1.07	0.23	–3.21	0.85	–0.10
GROMACS (MD)	–0.26	–0.13	0.62	0.03	–1.03
HOOMD-blue (MD)	–0.25	–0.08	0.59	0.99	0.53
MCCCS-MN (MC)	–0.22	–0.02	0.59	–0.84	0.15
GOMC (MC)	–0.24	–0.09	0.60	–0.82	0.28
Cassandra (MC)	–0.10	0.09	0.80	–0.22	–0.01

The maximum difference is found between LAMMPS and these four engines with a difference of 0.008 kJ/mol/molecule. The degree of these differences likely arise from unit conversions of force field parameters (specifically the LJ epsilon), as several of the engines used different internal units, or from well-known issues related to variations that will occur when the same operations are performed in different order. Tail corrections also agree very closely with maximum deviations for GROMACS, 0.2%. Using the energy breakdowns, electrostatics show the most variance and likewise source of engine discrepancies, which is due to the architecture-specific algorithms in place to break up this long-range force and optimize run times. This high level of agreement is compared to the work of Shirts et al.^36^ that examined implementation differences between the GROMACS, AMBER, LAMMPS, DESMOND, and CHARMM molecular simulation engines, finding 0.006% relative absolute potential energy deviation for all energy components, which agrees with the order of magnitude relative deviations found in this work.

In general, variations observed in the single point energy calculations can come from six key differences specific to the system initialization:

(1) the parameters and force field method converted from literature,
(2) the initial configuration for rigid or semiflexible molecules is not consistent with the force field parameters,
(3) the method or software that converts these inputs to simulation engine specific formats,
(4) specified precision differences in input files,
(5) differences or bugs in underlying algorithms in the simulation engines (e.g., fixed vs flexible bonds, different long-range solvers for electrostatics), and
(6) force field implementation in the engine control files.

MoSDeF directly addresses three of these sources of error. Source (1) is addressed through the use of a single force field file, which ensures every engine sources the same parameters. A common source of force field parameters and automated unit conversions for specific simulation engines greatly reduces the potential for errors due to unit conversions of parameters taken from the literature. Source (2) is partially addressed through mBuild and Foyer, which have modules to validate the methods used to atom type and initialize the topology. Lastly, source (3) is reduced because MoSDeF includes engine specific writers that are tested for a wide array of models, and any conversions are performed with well-defined methods.

The remaining initialization errors are not addressed by MoSDeF, and they are solely dependent on the choice of the simulation engine and their users. Due to the elimination of the first three error types, we are able to isolate these engine specific implementation differences in a controllable fashion and quantify their effects on practical replicability. Error type (4) from the input file precision is currently irreducible, since files have hard limits, while others have simply been capped by the software used to generate them. Specifically, GROMACS GRO 87 files used in this project are limited to 8 total characters (including the decimal point), where we note coordinates are defined in nm. In this study, GOMC uses PDB files, which have a total of 6 characters, including the decimal point with a maximum of 3 decimal places, defined in Å. While GOMC can also use double precision COOR files, COOR files are not currently supported by MoSDeF. The LAMMPS data files generated by MoSDeF utilize 6 decimals (units of Å), the HOOMD-blue GSD file is a binary format with 32 bit floating point precision (in user-defined units), the MCCCS-MN coordinate file provides 18 total digits (Å), and Cassandra XYZ files have 18 character precision. We note that the precision for energies are found to be to 5 significant figures, which is in line for the rounding found from the input file error source. Error type (5) is inherent to individual engines; some engines do not have support for a particular method, such as long-range treatments, make concessions on algorithm accuracy for speed, or have bugs in their methods that are, presumably, identified and addressed over time, and is a driving factor for open-source, community-contributed simulation codes. This source of error cannot be controlled for in the context of this work, but the results of more reproducible and comparable simulations should mitigate these errors, and some of these bugs were identified during the course of this work and discussed below. Finally, error type (6), the force field implementation, is caused by mistakes in the force field translation to the desired engine. This was the most difficult error to overcome as it required a cumbersome trial and error approach. Some of the lessons learned during this back and forth process are annotated below.

The following paragraph contains a list of issues that arose during initialization. Error type is given in parentheses.

(2) Configuration error: an issue with the initial configurations was uncovered within the PACKMOL software that mBuild uses to place molecules within the simulation box. The random seed that PACKMOL uses can be specified to reproduce the configuration, but that configuration can differ if the code was run on Mac OS vs Linux architectures. Bugs such as this are not identified unless rigorous calculations are performed on the system, due to the inherent assumption within MS that such minor differences will not affect MS output beyond statistical errors. Because this issue (https://github.com/m3g/packmol/issues/30) was not easily addressed in the PACKMOL software, a workaround was found in MoSDeF in which the initial configurations were generated and written to mBuild JSON files, which could be provided directly to all engines. These issues will inhibit full reproducibility. Additionally, similar errors have arisen in other work outside this scope; for example in the popular package OpenBabel, the code used to generate 3D structures from SMILES is stochastic (https://github.com/openbabel/openbabel/issues/1934).
(4) Conversion error: an initial single point calculation performed for Cassandra resulted in 0.0002% error for water–SPC/E, which was about 100× the error found across the other simulation engines. Upon further examination, the result was attributed to the rounding of the LJ σ from 0.316557 to 0.3166 nm. Likewise, incorrect conversion of the LJ ϵ in LAMMPS was inspected for methane–TraPPE. The TraPPE-UA force field specifies the LJ well depth in thermal units as ϵ/*k*_B_ = 148.0 K, but LAMMPS accepts only kcal/mol as the energy unit and with limited precision. The value used in LAMMPS converts to 148.001897 K, so the total potential energy was calculated in MCCCS-MN also using this precise but slightly incorrect ϵ value. The system energies for MCCCS-MN@148.0 K, MCCCS-MN@148.001897 K, and LAMMPS@148.001897 K (but in kcal/mol) are respectively 536736.674114, 536743.553773189, and 536743.553773 kJ/mol, which gives good evidence that incorrect conversion with limited precision of the LJ well depth is the major contributor for the inaccurate value obtained with LAMMPS at this level of precision.
(5) Algorithmic differences: at the start of the project, HOOMD-blue version 3 had not yet implemented a LJ long-range tail correction. Because HOOMD-blue was developed by the authors of this work, we were able to contribute this functionality to the engine. This feature was validated by comparing to the tail correction energies from other MoSDeF supported MS engines.
(5) Engine bugs: GROMACS encountered issues for GROMACS version 2020.6. For the steepest descent energy minimization method, there was a bug in the energy calculation routine. This is fixed in subsequent releases; the issue is currently linked to GitHub: https://gitlab.com/gromacs/gromacs/-/issues/4229).
(6) User error: for GROMACS and LAMMPS, the 1–4 nonbonded scaling factor for TraPPE-UA force field was not specified in the input files to those engines. As such, control file templates had to be modified to include this specification, which was noticed when these molecules had differing nonbonded energies. This issue was addressed in Foyer in https://github.com/mosdef-hub/foyer/pull/489.

The congruence of such results, across three MD and three MC engines, is an initial attestation to the TRUE^37^ nature of MoSDeF. We have demonstrated that the usage of a common initialization routine via MoSDeF can minimize deviations between the force field implementations in the different engines. All single-point energy deviations appear to be on the scale of those observed in other studies (e.g., Shirts et al.^36^). In this way, we have largely eliminated all major errors in the initial configuration and model setup. This reduction in error sources allows us to focus on possible differences that are inherent to the simulation methods and algorithms themselves.

### 3.2 Methane–TraPPE

Density data are gathered across six engines for methane–TraPPE (all molecule density values are reported in Tables S9–S11). This TraPPE-UA model involves only a LJ potential, which allows for a simple comparison across the engines. The lack of electrostatics for the model removes the *k*-space calculations for the long-range force, which, as noted in the single point energy analysis, is a source of implementation variance across the engines, and has been observed in other work to be the most significant contribution to energetic differences.^36^ The calculated density (in kg/m^3^) agrees to three significant figures across all engines (Figure 2). The maximum deviation from the mean value is found for Cassandra which is 0.03%. We note that the error bars (95% CI) for the MC engines are 2–4 times larger than those of the MD engines due to fewer overall uncorrelated samples (note this study did not optimize MC runtime parameters to improve sampling efficiency), which is consistent across all molecules studied. While all engines do not agree within their 95% CI, the MD and MC engines separately fall within the 95% CI of their respective groups. A block difference of 0.20 kg/m^3^ exists between the average densities of the MD and MC engines.

**Figure 2:**
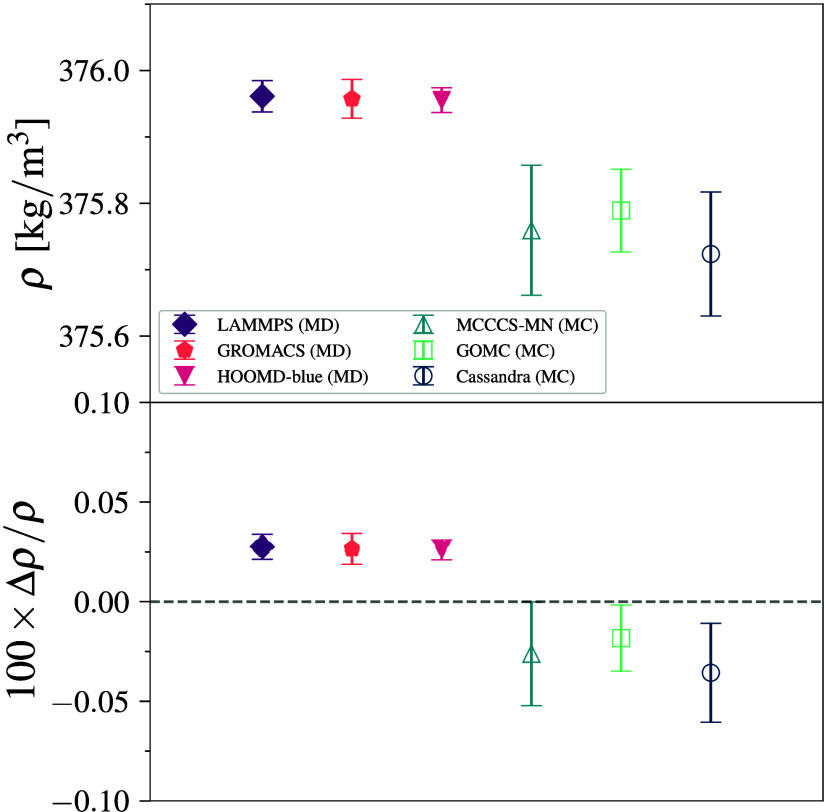
Density (top) and deviation from the mean density (bottom) for methane–TraPPE simulations at 140 K and 1318 kPa. Error bars denote 95% CI.

Apart from the principal distinction in how the configurational space is sampled, several subtle differences exist between MD and MC simulations that can impact their respective outcomes. These differences include but are not limited to

(1) Effect of time step in MD simulations^78^ and move probabilities in MC simulations.^79^ Different choices can alter results in the simulation methods and require trade-offs between efficiency and accuracy.
(2) For a general system with *N* particles (e.g., methane–TraPPE in this study), 3*N* degrees of freedom (DOF) are assigned in MC simulations, whereas MD assigns 3*N* – 3 DOF due to the assumption that the center of mass of the system remains stationary. This difference in the system DOF can lead to slightly different ensembles being sampled in the two simulation methods.
(3) The hard cutoff of the LJ potential at a finite *r* introduces a discontinuity in the force and its derivatives. While the MC method is unaffected by the discontinuity in force, it creates algorithmic difficulties for MD, since MD is a numerical solution to the equations of motion with error *O*(δ*t* ^2^) *with the assumption that the potential energy is infinitely differentiable*, where δ*t* is the time step used in the MD simulation.

We conduct a comprehensive investigation of these differences to pinpoint the underlying causes for the disparities observed in the results between MD and MC simulations for methane–TraPPE. First, we test the effect of time step in our simulations by conducting additional MD (LAMMPS) simulations for the methane system using a time step of 0.5 fs, which is four times smaller than the time step value we used for our original methane LAMMPS simulations. We obtain statistically similar results (see SI) for the two time steps, implying that a time step of 2 fs for methane simulations is appropriate for our purposes. Furthermore, we also perform simulations for a system of *N* = 1728 particles interacting via the Weeks–Chandler–Anderson (WCA) potential^80^ in MD and MC (see SI for details). Both the WCA energy and force are 0 at the cutoff radius, which removes the long-range pressure correction from consideration and decreases the error due to discontinuous potentials compared to a LJ potential with a hard cutoff. We get matching results for the density between MD and MC, which indicates that the temperature and pressure differences arising from different DOF treatments do not result in significant density differences.

Subsequently, we proceeded to investigate the effects of cutoff and tail corrections. To this end, we performed MD and MC simulations in the *NpT* ensemble for methane employing the TraPPE-UA force field parameters. As described in Section 3.1, there are small differences between the force field parameters used in different simulation engines, because of unit conversions. Since we wanted to analyze the difference in results between MD and MC, we decided to use the adjusted methane ϵ/*k*_B_ value of 148.001897 K instead of 148.0 K for MCCCS-MN simulations, as it is easier to modify the MCCCS-MN parameters to match LAMMPS due to the input file standards. We consider 12 distinct values of the cutoff radius (see Table 4) which correspond to distance values where the methane–methane radial distribution function (RDF) attains a maximum, a minimum, or a value of unity. An 1800-molecule methane system was utilized, ensuring that the box length remained greater than twice the cutoff distance at all of the *r*_cut_ values tested. We conducted 256 independent simulations to investigate on a finer scale. All other simulation settings were consistent with those reported in Section 3.2. The density results corresponding to different *r*_cut_ values are presented in Table 4.

**Table 4:** Effect of *r*_cut_ on Methane Density Computed Using MCCCS-MN (ρ_MC_) and LAMMPS (ρ_MD_)­^a^

	ρ_MC_ [kg/m^3^]		ρ_MD_ [kg/m^3^]				
*r*_cut_ [Å]	value	*u*	value	*u*	$\frac{\left(\rho_{\mathrm{MD}}-\rho_{\mathrm{MC}}\right)\times 100}{\rho_{\mathrm{MC}}}$	AEMC	AEMD
10.7	375.310	0.020	375.965	0.011	0.18	–0.15	0.018
11.4	375.948	0.021	375.872	0.011	0.021	0.019	–0.0065
12.5	376.100	0.019	375.981	0.011	0.032	0.060	0.023
13.2	375.858	0.021	375.980	0.011	0.032	–0.0046	0.022
14.3	375.805	0.021	375.918	0.012	0.029	–0.019	0.0057
15.1	375.890	0.021	375.907	0.011	0.0053	0.0038	0.0030
16.0	375.908	0.020	375.912	0.011	0.0010	0.0086	0.0042
16.9	375.882	0.021	375.889	0.011	0.0027	0.0017	–0.0020
17.0	375.873	0.019	375.911	0.011	0.011	–0.00069	0.0039
17.9	375.882	0.021	375.889	0.011	0.0027	0.0017	–0.0020
18.0	375.877	0.020	375.906	0.011	0.0080	0.00043	0.0026
24.0	375.876	0.020	375.896	0.011	0.0053	0.000	0.000

a A system of 1800 methane molecules was used for the simulations. The uncertainties, denoted by *u*, represent the 95% confidence interval. AEMC and AEMD refer to the asymptotic errors in density values from MC and MD simulations, respectively, at the *r*_cut_ value compared to simulations with a 24 Å cutoff. AEMC and AEMD are computed using the expressions $\frac{\left(\rho_{\mathrm{MC}}-{\rho_{\mathrm{MC}}|}_{r_{\mathrm{cut}}=24Å}\right)\times 100}{{\rho_{\mathrm{MC}}|}_{r_{\mathrm{cut}}=24Å}}$ and $\frac{\left(\rho_{\mathrm{MD}}-{\rho_{\mathrm{MD}}|}_{r_{\mathrm{cut}}=24Å}\right)\times 100}{{\rho_{\mathrm{MD}}|}_{r_{\mathrm{cut}}=24Å}}$ , respectively.

As seen in Figure 3, at higher values of *r*_cut_, MD and MC converge and produce statistically indistinguishable results. As the value of *r*_cut_ is increased, the magnitude of discontinuity in the LJ potential at *r*_cut_ decreases. Hence, we attribute the observed density disparity between MD and MC simulations for methane in Section 3.2 to the problems arising from the utilization of a finite cutoff distance. Moreover, we note that although increasing the value of *r*_cut_ may appear to yield more consistent results, the choice of *r*_cut_ is an inherent component of the force field. Altering this parameter would imply modification of the force field itself. Hence, when reproducing and comparing results, meticulous attention must be paid to ensure consistency and validity when employing the force field.

**Figure 3:**
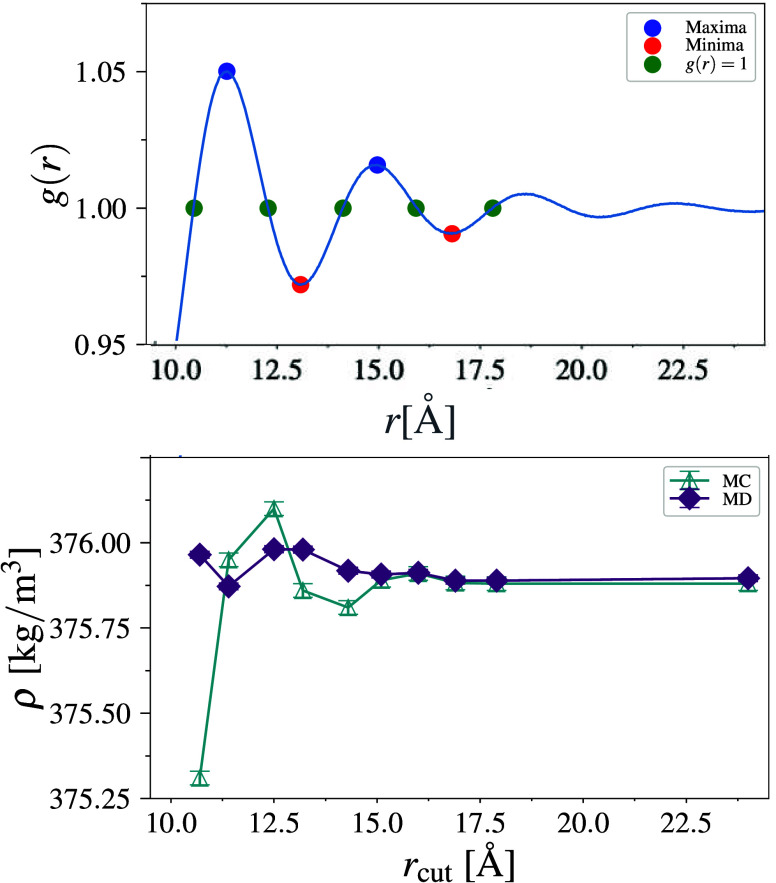
(top) Zoomed-in view of the methane–methane RDF (*g*(*r*)) for the 1800-molecule methane system (*r*_cut_ = 24 Å). The peak at 11.4 Å is the third peak in the RDF. Blue, red, and green circles show the points where *g*(*r*) is maximum, minimum, and attains a value of unity, respectively. (bottom) Variation of methane density with *r*_cut_ for MD (purple diamonds) and MC (blue up triangles). Error bars show a 95% CI.

### 3.3 Pentane–TraPPE

Pentane–TraPPE, which includes bonded restraints in the system, also shows close agreement between engines with results similar to those observed for methane–TraPPE (see Figure 4a). All engines show agreement within 0.2%, where again, MD engines predict slightly higher values than MC with a difference between the average values of MD and MC of 0.7 kg/m^3^. We note that the MD engines treat the molecules as flexible chains with constraint algorithms to fix the bond lengths, whereas the MC engines do not alter bond lengths (unless explicitly specified). Given that the relative error is about four times larger than that for methane (while *r*_cut_ = 14 Å is used for both), it appears that differences between fixed and constrained bond lengths are significant, even for united-atom models interacting via LJ potentials. We note that LAMMPS data are not included because its current constraint algorithm cannot be used for bonds in a chain of atoms.

**Figure 4:**
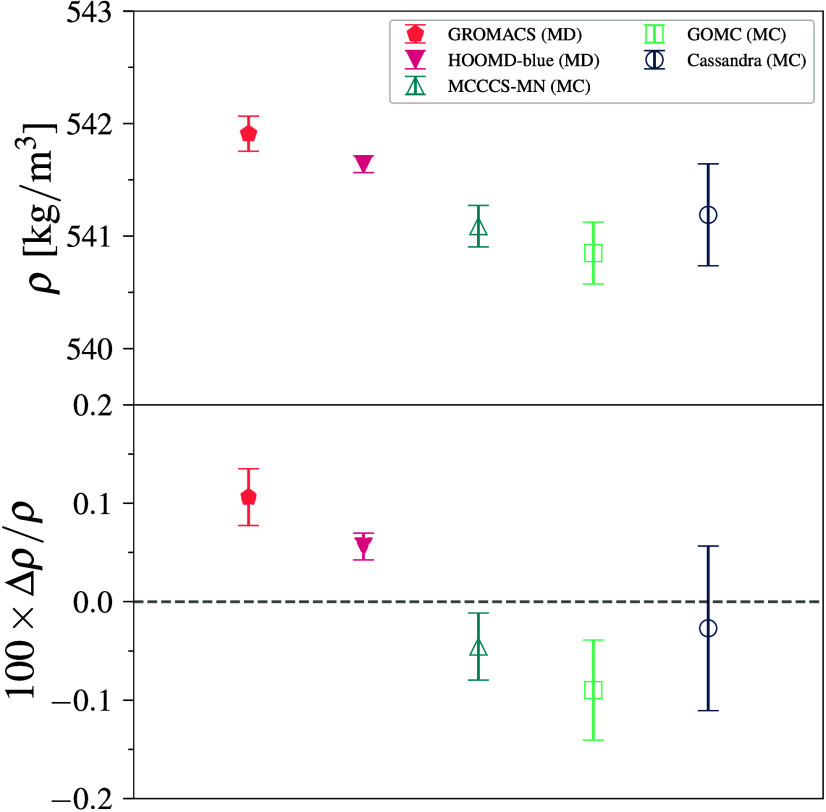
Density (top) and deviation from the mean density (bottom) for pentane–TraPPE simulations at 372 K and 1402 kPa. Error bars denote 95% CI.

### 3.4 Benzene–TraPPE

Benzene–TraPPE is treated as a fully rigid molecule (with bond lengths, bending, and dihedral angles), and we find densities agreeing to within 0.2%. We see that the three MC engines all agree within the 95% CI, but the two MD engines do not (Figure 5). We note that GROMACS data are not included, as the main branch of the code does not support rigid bodies. The disagreement between MD engines is attributed to a difference in the way that HOOMD-blue and LAMMPS calculate the virial contribution to pressure. There are two equivalent methods for this computation, the atomic (implemented in LAMMPS) and molecular virial (implemented in HOOMD-blue), which are viewed, respectively, from the contribution of the constraint force on the individual atoms or as a net force on the rigid molecule center of mass. While shown to be equivalent in theory, implementation differences can still lead to a small discrepancy.^81^ We also note that the LAMMPS simulations result in benzene molecules showing larger deviations from planarity. The difference between updated HOOMD-blue and LAMMPS is about 0.21% and was only identifiable by precise control of the rest of the simulation inputs and a direct consequence of how MoSDeF can enable cross-engine validation.

**Figure 5:**
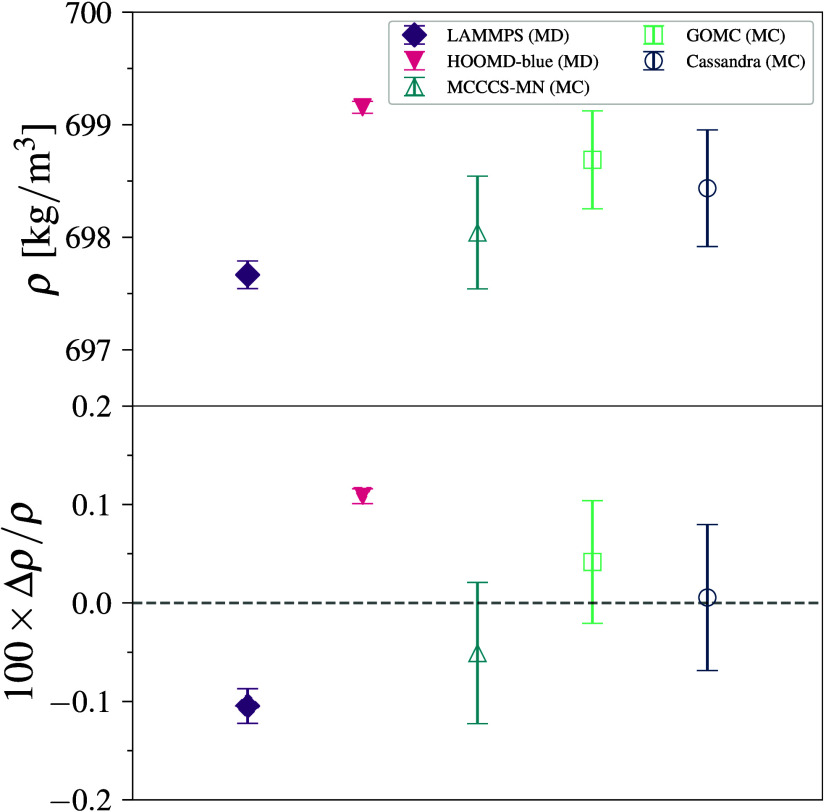
Density (top) and deviation from mean density (bottom) for benzene–TraPPE simulations at 450 K and 2260 kPa. Error bars denote 95% CI.

### 3.5 Water–SPC/E

Water–SPC/E simulations are done at three different temperature points to capture the thermal expansivity of the water model. The results are reported in Figure 6 (and Table S9) and show excellent agreement to within 0.07%. It is interesting that the relative deviations between simulation engines observed for the specific densities are smaller for water than for *n*-pentane and benzene. That is, the larger single-point errors for the long-range electrostatics are less of a problem than conformational flexibility (*n*-pentane) and more complex rigid shapes (benzene). The SPC/E model also specifies a rigid molecule, which helps reduce the short time scale bond oscillations that can cause barostat sampling error. The MD and MC engines agree among each other within the 95% CI.

**Figure 6:**
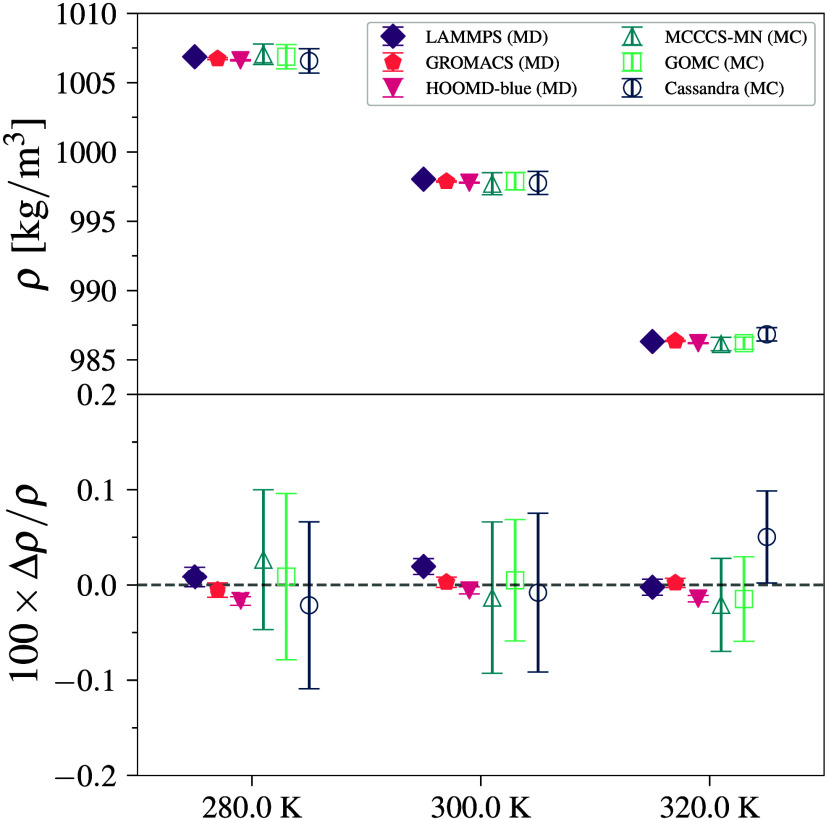
Density (top) and deviation from mean density (bottom) for water–SPC/E simulations at 280, 300, and 320 K and at 101.325 kPa. Error bars denote 95% CI.

One of the lessons learned during the single-point calculations in Section 3.1 was that MCCCS-MN used parameters obtained from a different source compared to those of the rest of the engines. All engines except MCCCS-MN converted their parameters from a Foyer force field, which was using the GROMACS provided SPC/E parameters.^82^ Meanwhile, MCCCS-MN used parameters from NIST^83^ and assumed equivalency. The resultant single point energy indicated that, while LAMMPS, GROMACS, HOOMD-blue, GOMC, and Cassandra agreed with less than 0.0001% error, MCCCS-MN showed a 0.001% error; an order of magnitude higher difference. This observation led to a closer investigation into the sources of force field parameters. We found some interesting discrepancies between different versions of water–SPC/E parameters distributed across the community, which are summarized in Table 5. Most notably, none of the packages had exact matches, even with rounding to four significant figures (original/unrounded significant figure shown in Table 5), to the original work published by Berendsen et al.^48^ While NIST reported the most accurate ϵ to 6 significant figures, the LAMMPS-provided version has the accuracy to only one significant figure. The original values are given in a different form of the LJ equation, with *A* and *B* values of 0.37122 (kJ/mol)^1/6^ and 0.3428 (kJ/mol)^1/12^ ·nm, respectively. Conversion from the original formula to a more standard form is nontrivial and introduces error associated with thermal units. These specific conversions involve physical constants such as Avogadro and Boltzmann constants, which are not consistently rounded or even necessarily reported from each source. Notably, the five engines that use GROMACS distributed parameters and MCCCS-MN, which uses the NIST distributed parameters, have values that agreed to 4 decimal places and fall within the 95% CIs at all temperatures. This consistency provides strong evidence that the less than 0.01% difference in LJ parameters does not cause a greater variance than the ensemble sampling for this model. The thoroughness of the NIST parameters is paralleled with the fact that they also report the exact values for the constants used to obtain them, unlike the other sources. In order to further probe the possible effects of using different LJ parameters currently available, we simulate water–SPC/E using the GROMACS engine with parameters obtained from the Berendsen et al., LAMMPS, and GROMACS. Systems with LAMMPS distributed parameters give the most dissimilar values, which can be attributed to the fact that these values are reported originally in kcal/mol and were presumably converted from OPENMM as they appear to share a similar difference.Figure 7 shows results from different SPC/E parameters. LAMMPS parameters yield significant deviations from the original force field, which if left unchecked could be a rising issue in the field. These observations underscore the importance for the community to publish transparent and well documented conversions where readers can clearly understand how parameters used in the specific simulation engine are obtained. Reporting actual simulation parameters along with the DOI of their original source, which can be done via tools such as Foyer, should be an academic standard that would mitigate misrepresentation of the models used across MS.

**Table 5:** Comparison of Published Parameters for the Water–SPC/E Lennard-Jones Model to the Original Source^a^

source	σ [Å]	ϵ [kJ/mol]	ϵ [K]
original SF^a^	3.166	0.6502	78.20
original HP^b^	3.1655578901998814	0.6501695808187480	78.1974266622128301
NIST	3.16555789	0.6501696178	78.19743111^c^
GROMACS	3.16557	0.650194^c^	78.2004
OPENMM	3.1657195050398818	0.6497752^c^	78.14999
LAMMPS	3.166	0.6498	78.15

a Underline indicates the last decimal place of agreement. σ is typically given in nm or Å and is trivially converted. *ϵ* is shown under both common conversion methods, with Kelvin (K) and kJ/mol units, which requires Boltzmann’s and Avogadro’s constant, for which we have used 1.380649 x 10^-23^ J/K and 6.02214076 x 10^23^ respectively.

b Significant figures (SF) rounding to 4 places converted from Berendsen et al.^48^

c High precision (HP) value converted from Berendsen et al.^48^

d Units in which the source provides.

**Figure 7:**
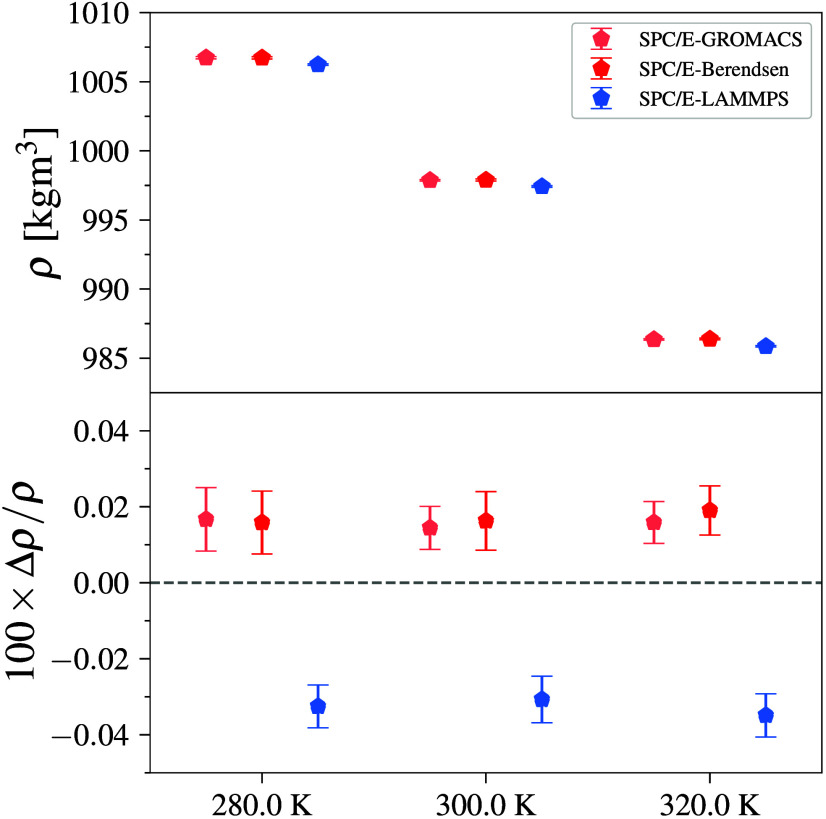
Density (top) and deviation from mean density (bottom) for water–SPC/E simulations using the GROMACS engine with parameters reported from different sources (GROMACS, Berendsen original high precision (HP), LAMMPS) at 280, 300, and 320 K and at 101.325 kPa. Error bars denote 95% CI.

### 3.6 Ethanol–OPLS

Similar to the water–SPC/E model, the ethanol–OPLS model includes partial charges and is tested at three different temperatures, namely, 280, 300, and 320 K (Figure 8). The MD engines yield statistically significantly higher densities by 3 to 5 kg/m^3^ than the MC engines. The relative error between the engines increases from 0.3% to 0.4% as the temperature increases. Interestingly, the data for the three MD engines show significant deviations among themselves, whereas the MC data agree with each other. Ethanol–OPLS is commonly modeled with fixed bond lengths in MC but with flexible bonds governed by harmonic potentials in MD. While this modification in bond treatment is considered an acceptable practice in MS since some engines implement only one of constrained or flexible bonds, the effect appears to be significant for the ethanol–OPLS models.^14,16,84^

**Figure 8:**
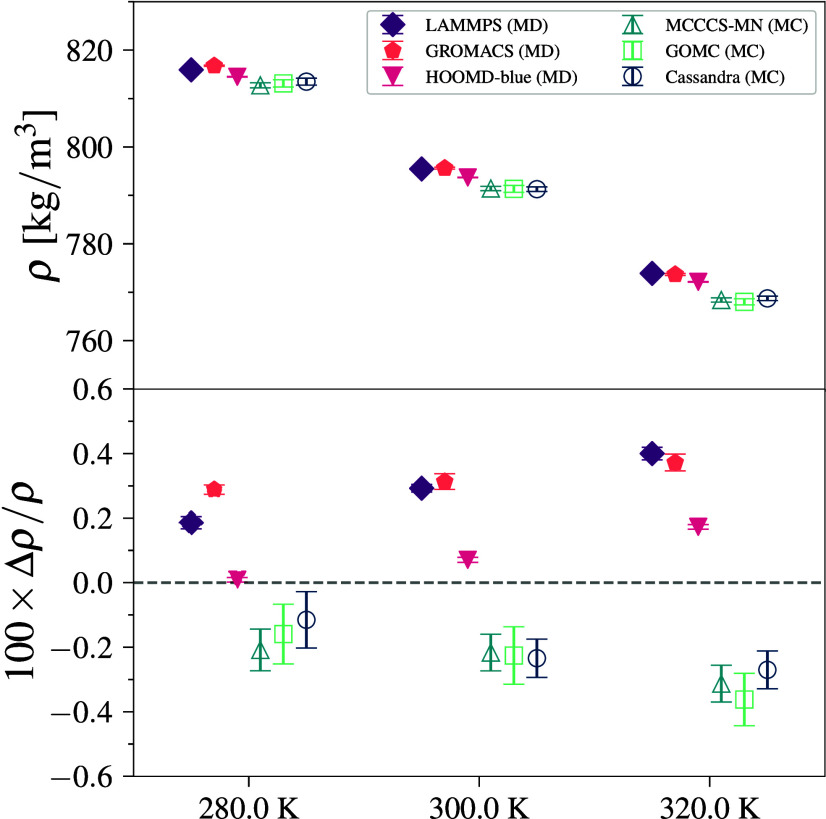
Density (top) and deviation from mean density (bottom) for ethanol–OPLS simulations at 280, 300, and 320 K and at 101.325 kPa. Error bars denote 95% CI.

Figure 8 highlights a relative error in density of approximately 0.4% between MD and MC engines compared to more statistically similar results within MD and MC approaches. The underlying factors possibly causing this difference between the two simulation algorithms are discussed in Section 3.2, but they are found to be smaller than the 0.4% difference observed here. To understand the contribution of bond treatment, i.e., rigid or flexible, in MD and MC methods, we performed additional simulations, aiming to match the ethanol–OPLS implementation in both simulation methods.

Our approach involves modeling ethanol–OPLS systems using MC simulations with all bonds treated as rigid and MD simulations with the bond force constant derived from the OPLS-AA force field, allowing the bonds to be flexible. For the new set of simulations, we use a fully flexible ethanol–OPLS model in MCCCS-MN and a partially rigid ethanol–OPLS model in LAMMPS. In the LAMMPS simulations, we keep all bonds flexible, except for the O–H bond, which we constrain at the equilibrium O–H bond length from the ethanol–OPLS force field using the SHAKE algorithm. This allows us to investigate the effect of bond flexibility on the density and structure differences between MD and MC simulations while keeping the implementation of the ethanol–OPLS force field as consistent as possible between the two simulation algorithms.

Figure 9 shows the density values for the four different simulations, namely, fully flexible ethanol–OPLS in LAMMPS (LAMMPS-flex), partially rigid bond ethanol in LAMMPS (LAMMPS-fixOH), fully flexible ethanol in MCCCS-MN (MCCCS-MN-flex), and rigid bond ethanol in MCCCS-MN (MCCCS-MN-fix) for three temperatures (Table S12 includes the raw data). For both simulation engines, the density is higher for the fully flexible bonds model as compared to the partially constrained or fully fixed bonds models. Agreement between LAMMPS-fixOH and MCCCS-MN-fix results is observed at 280 K with the relative error being <0.2%, which is nearly half of the relative error obtained in the original ethanol–OPLS simulations. Moreover, the difference between LAMMPS and MCCCS-MN simulations also decreases (<0.1%) if a flexible bonds model is used in both simulation engines. This indicates that the major contributor to the difference we observed between MD and MC ethanol–OPLS results is coming from bond treatments. It should also be noted that the MCCCS-MN-flex data agree within 0.1% with the HOOMD-blue data for the same model.

**Figure 9:**
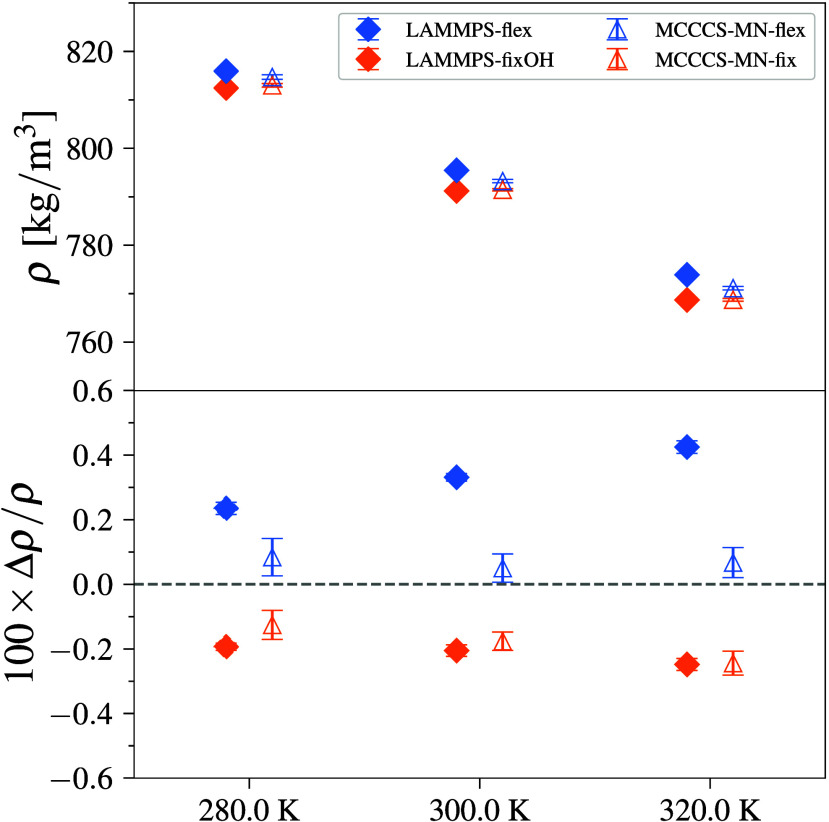
Density (top) and deviation from mean density (bottom) for ethanol–OPLS simulations in LAMMPS-flex (blue solid diamonds), LAMMPS-fixOH (orange solid diamonds), MCCCS-MN-flex (blue triangles), and MCCCS-MN-fix (orange triangles) at 280, 300, and 320 K and at 101.325 kPa. Error bars denote 95% CI.

To understand the differences in the structures of flexible and fixed bond ethanol–OPLS simulations, we report the hydrogen bond (HB) counts in Figure 10, the O–H bond length distributions for these systems in Figures S4–S6, the RDFs in Figure S7, and the cumulative distribution functions (CDFs) in Figure S8. We apply an elliptical hydrogen bond criteria, requiring the O–O distance and the donor–hydrogen–acceptor angle to fall within an elliptical boundary.^85,86^ Derived from radial-angular distribution functions (Figure S9), the ellipse is centered at 2.75 Å and 180°, with a half width of 0.5 Å and 50°. Conventionally, MC simulations constrain all bonds, even if the bonds are defined as flexible in the original force field. The equilibrium O–H bond length as defined in the OPLS-AA force field is 0.945 Å. However, when the bond is made flexible in the MCCCS-MN-flex model, a positive shift of 0.018 Å in the mean O–H bond length is observed (see top right of Figure 10); this lengthening of the bond leads to an increased bond dipole. The calculated number of hydrogen bonds is much higher in flexible ethanol–OPLS simulations as compared to the fixed bonds models, regardless of being simulated using MD or MC. Furthermore, the flexible bond models have a higher first peak in the O–O RDF and a slightly larger value of the CDF (e.g., at 3.25 Å, the *n*(*r*) value is larger by 0.04 which is consistent with an increase in 20 = 0.04 × 500 hydrogen bonds). This increase in packing density can be attributed to the ability of the O–H bond to stretch and make more and stronger hydrogen bonds. These findings highlight the importance of exact force field implementation and the effect of using fixed bonds in MC simulations. For MD, the use of SHAKE constraints on the OH bond of the ethanol–OPLS model affected the density by about 0.5% at 300 K. This comparison provides insights into the factors that contribute to the observed density difference for hydrogen-bonding systems between MD and MC simulation engines and underscores the need for careful consideration of these factors in MS research.

**Figure 10:**
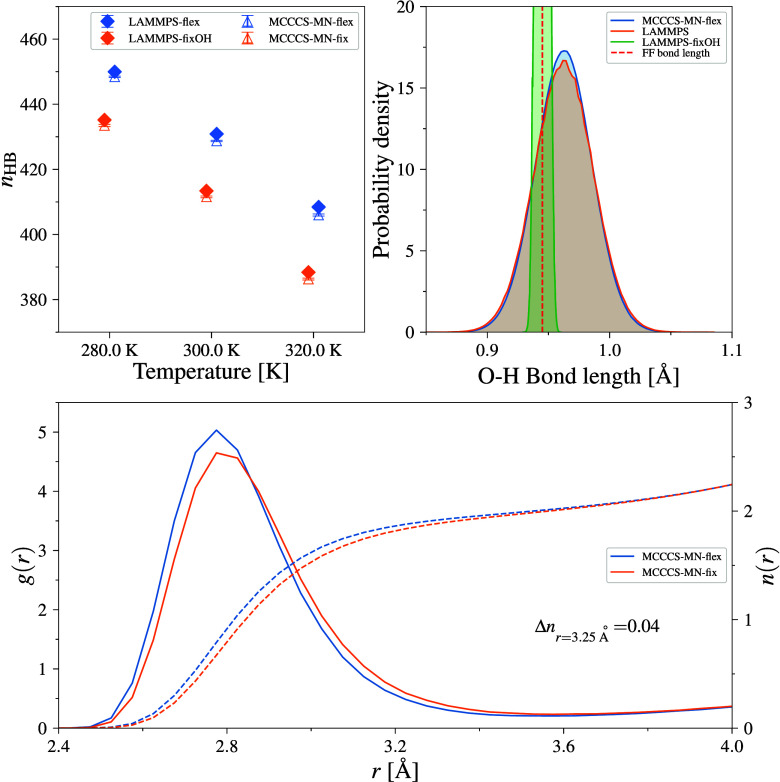
(top left) Number of hydrogen bonds (*n*_HB_) for ethanol–OPLS simulations for a 500-molecule system in LAMMPS-flex (blue solid diamonds), LAMMPS-fixOH (orange solid diamonds), MCCCS-MN-flex (blue triangles), and MCCCS-MN-fix (orange triangles) at 280, 300, and 320 K, all at 101.325 kPa. Error bars denote 95% CI. (top right) O–H bond length distribution for ethanol–OPLS simulations in MCCCS-MN-flex (blue), LAMMPS-flex (orange), and LAMMPS-fixOH (green) at 280 K and 101.325 kPa. Dashed vertical red line denotes the equilibrium O–H bond length from the OPLS-AA force field. (bottom) O–O RDF (*g*(*r*)) (solid lines) and CDF (*n*(*r*)) (dashed lines) for MCCCS-MN-fix and MCCCS-MN-flex simulations at 280 K and 101.325 kPa. MCCCS-MN-flex and MCCCS-MN-fix results are shown in blue and orange, respectively.

### 3.7 Effect of Long-Range LJ Correction and Cutoff Treatment

The LJ long-range correction (LRC) is a method to account (approximately) for interactions between sites separated by distances larger than the cutoff distance for which the potential energies and, if applicable, the forces are not calculated explicitly. There are many methods employed in the literature, and their impact on predictions is, unfortunately, sometimes considered to be negligible and is ignored. Not all implementations are available in all simulation engines. For this work, we added energy-pressure tail corrections to HOOMD-blue. We note that the LRC simulations carried out with the HOOMD-blue software used version v3 Beta14, whereas the results reported in preceding sections were based on HOOMD-blue v4. While it would have been ideal to maintain uniformity by employing the same software version, the ability to cross-engine compare single-point energies revealed no disparities between the two versions. Hence, a decision was made to utilize the beta v3 results to minimize simulation reruns.

In order to exactly compare force field implementations across engines, it is important to understand how choosing the LRC compares to other engine or model differences. We choose to consider a simple hard-cutoff (i.e., interactions beyond the cutoff are ignored), a shifted cutoff (i.e., interactions beyond the cutoff are ignored, and the site–site LJ potential energy is shifted up by the negative of its value at *r*_cut_, which does not affect the forces), and an energy–pressure tail correction to the LJ forces, which are available across the engines in this study and are applied to both methane–TraPPE (Table S13) and water–SPC/E (Table S14). The hard-cutoff showed a clear split between the MD and MC implementations (seeFigure [Fig fig11] and Figure 12). This difference is caused by the MD engines not properly accounting for the discontinuity in energy at the cutoff distance, whereas the MC engines account for the energy jump in the acceptance rule. This leads to the MD engines erroneously predicting the same density values for the shifted cutoff and hard cutoff approaches, whereas the MC engines yield density increases of 1.0 and 0.6% for methane–TraPPE and water–SPC/E when switching from shifted to hard cutoff (because there is a favorable energetic gain when molecules move to within the cutoff). Both the energy-pressure tail and shift cutoff are handled more uniformly across MD and MC; the energy-pressure with tail correction was used for the other simulations in this work because the force fields considered in this work had been derived with this LRC.^46,48,49^ It should be noted that we also selected consistent time steps for the MD engines, comparing Weeks–Chandler–Anderson potentials^80^ in Table S15, which has continuous forces at the cutoffs, and comparing LAMMPS TraPPE-UA methane simulations at 2 and 0.5 fs time steps in Table S16.

**Figure 11:**
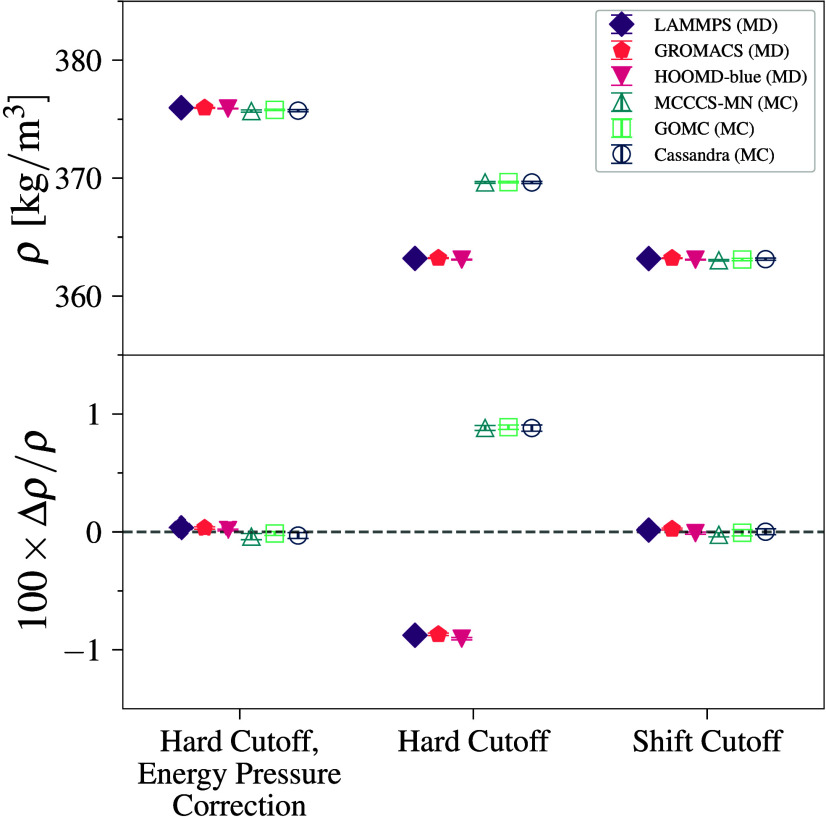
Effect of cutoff and long range correction on methane–TraPPE density at 140 K and 1318 kPa.

**Figure 12:**
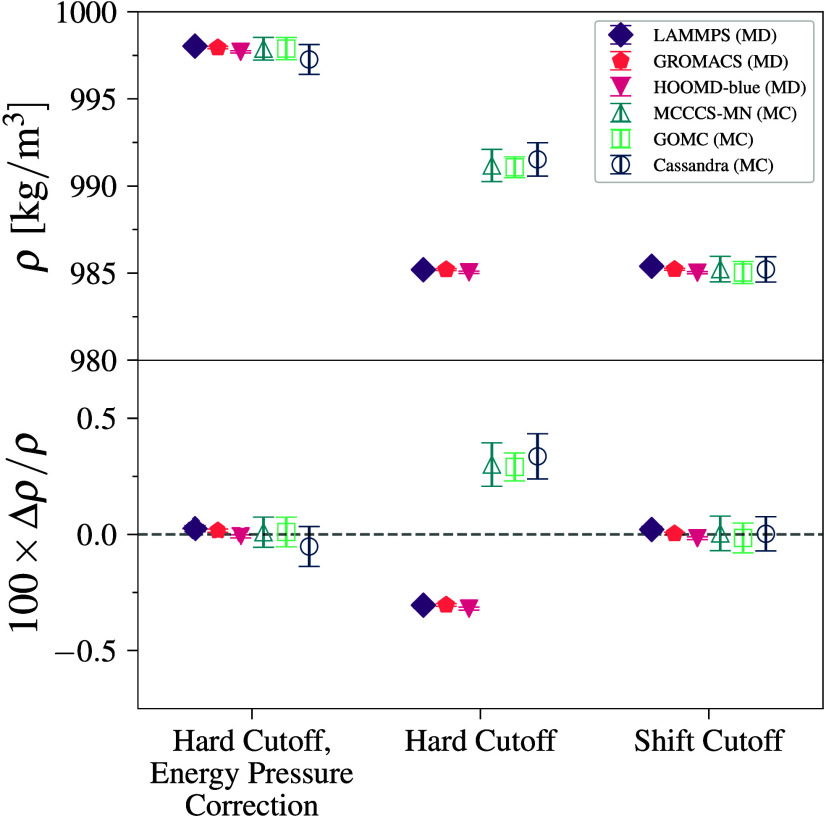
Effect of cutoff and long range correction on water–SPC/E density at 300 K and 101.325 kPa.

## 4 Discussion

To properly represent practical reproducibility and quantify the utility of tools such as MoSDeF to achieve TRUE simulations,^37^ it is important to understand the different ways that errors in simulation results can be compared. Figure 13 shows kernel density violin plots of the density relative error (eq 1) for the simulations considered in this work, referred to as MoSDeF systems/simulations. The plots include three rows, showing three possible grouping methodologies for generating the relative errors, and each plot is a probability density curve, with error bars indicating the minimum and maximum relative errors. We note that for both ethanol–OPLS and water–SPC/E, there are three temperature state points that are simulated and overlaid into the single probability density for the displayed models. The top row of Figure 13 takes each simulation and treats it as an individual sample to compare to the model mean; this grouping focuses on the relative error spread on a simulation-to-simulation basis. For example, the methane-UA density plot includes ninety-six individual simulations, with 16 replicate simulations for each of the six engines. Each of the ninety-six simulations are compared to the overall mean of the full set of 96 to find the relative error. Using this grouping method, the maximum deviation is 0.7% for ethanol–OPLS, which is the most complex model studied. However, this top row of results shows that some molecules, including ethanol–OPLS, have bimodal distributions, presumably resulting from the use of three MD and three MC engines. To view the distributions if we control for this, the middle plots show simulations compared only within their MD or MC groupings for the relative error calculation. In this instance, 48 methane-UA simulations are compared against the MD group mean, and the same is done for the other 48 MC simulations to generate the grouped relative deviations. The bimodal split due to the MD/MC deviations, which is most noticeable in the ethanol–OPLS model, is reduced but not fully eliminated. Finally, the results can be broken down by comparing only the average of the 16 replicates from each engine to the average across all engines, which gives an idea of the replicability across engines. A reduction of 3–4× in the errors is observed, which is expected given the $\sqrt{n_{\mathrm{replicates}}}$ dependence of sampling error. Most notably across these methods of measuring error, as model complexity increases from left to right, the actual relative error does not rise dramatically, which serves as evidence that MoSDeF has aided users in reducing the number of mistakes during these complex implementations.

**Figure 13:**
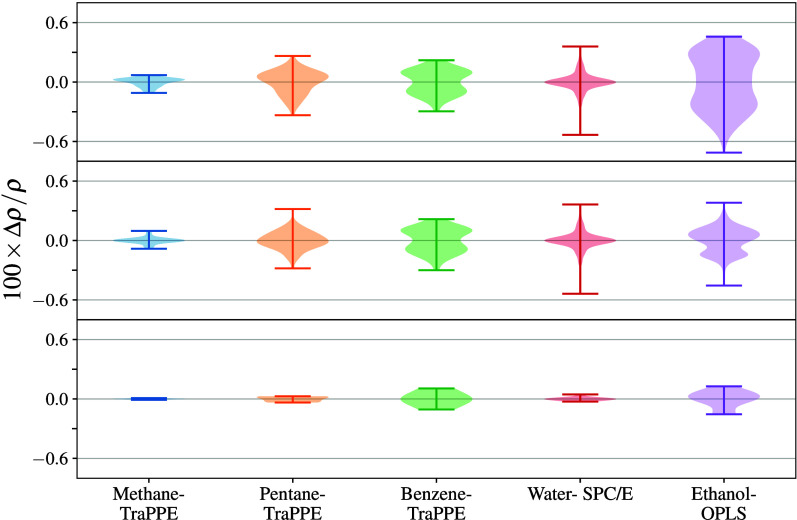
MoSDeF simulated molecule comparison with distributions of different relative error groupings. Groupings are (top) errors of the mean of each engine value calculated from the 16-replicate means, (middle) errors of each individual simulation compared with the mean of the MD or MC densities, and (bottom) errors of the individual simulations with the total mean for the molecule.

Using the methods of relative error on a per simulation basis, a head-to-head comparison can be made with the simulation data reported in the RR study. Figure 14 breaks it down by model types, with the molecules to the left of the dashed line representing models studied in the RR study and those to the right of the dashed line representing MoSDeF models used in this work. In both cases, the relative errors were calculated by their state point groupings. The top plot shows TraPPE-UA hydrocarbon models; the middle plot compares different models with rigid bonds and partial charges, OPLS-AA and SPC/E, and the bottom plot shows fully flexible model OPLS-AA models, where the MoSDeF ethanol–OPLS is an all-atom molecule and the RR molecules are united-atom. Figures S10–S13 show the scatter plots for the density deviations used to generate these violin plots.

**Figure 14:**
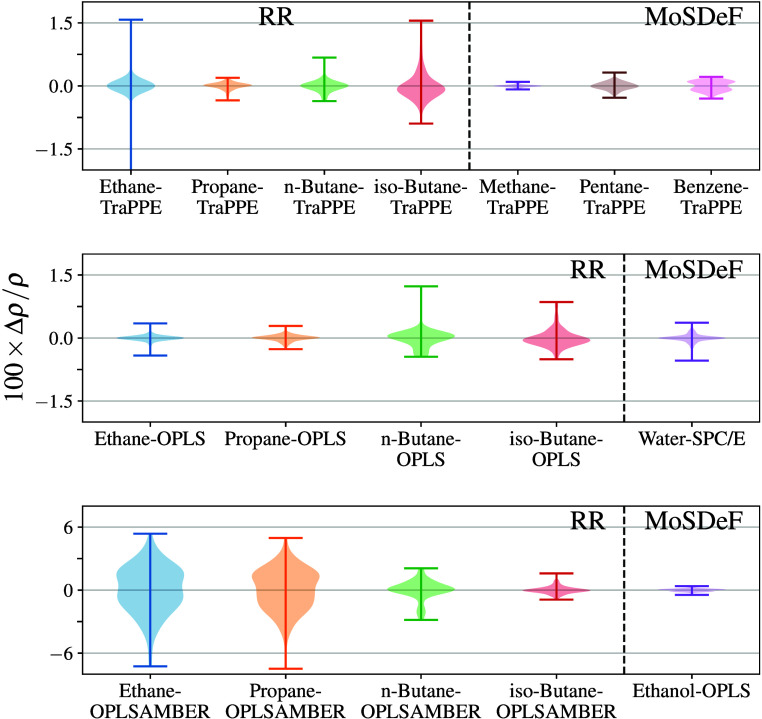
Round Robin paper (left of dotted vertical lines) relative errors compared to MoSDeF simulations done in this work (right of dotted vertical lines). Comparing models of (top) rigid model TraPPE-UA force fields, (middle) rigid model OPLS-AA to SPC/E force fields, and (bottom) flexible model OPLSAMBER to OPLS-AA force field.

After eliminating the clear outlier user-errors noted in the RR study, the maximum RR error was 7.5% for ethane–OPLSAMBER and the maximum MoSDeF error was 0.7% for ethanol–OPLS. To faithfully compare the results, outliers from the RR study (i.e., those with relative errors above 10%) were discarded since those simulations were obviously implemented incorrectly. This included the ms2­(KL) and GROMACS­(KL) engine implementations for ethane–TraPPE, which reported densities at 298 K and 5 MPa with extremely large relative errors of −80% and −20%, respectively. These two simulation sources also showed high relative error in other cases (e.g., 8% for ethane–OPLS at 298 K and 5 MPa) such that it was deemed sensible to remove this set of results from the ethane study entirely. Additionally, the entirety of the 5 MPa at 298 K state point simulations was dropped for ethane–OPLSAMBER, as an accurate density could not be ascertained due to the extreme disagreement across all 12 simulations: 97.84, 121.17, 203.17, 306.62, 369.21, 375.37, 387.10, 398.69, 399.15, 399.50, 402.00, and 418.90 kg/m^3^. The source of the discrepancies of the disagreement is not discussed in the RR paper, as the only pressure shown in the main text is at 41 MPa, and therefore, all data points were excluded in order to reduce the errors for ethane–OPLSAMBER. We note that the precise outliers to be dismissed are subject to interpretation. We do not wish to misrepresent the relative error deviation, where each individual value included influences every data point, since the overall mean can be shifted by large outliers. However, even with reasonable outlier removal for the RR work, the MoSDeF simulations fall on the same spectrum as the results for the best RR molecules and well within the error band for the least accurate RR molecules.

Figure 15 gives a visualization of all data for the RR and MoSDeF simulations. Each simulation is compared to its state point average, and the all-model distributions of these relative errors are analyzed. The maximum values of the extrema closely correlate with the central grouping of the RR results; within the MoSDeF maximum deviation cutoff of ±0.54%, there are 1854 RR simulations compared to 246 simulations that were outside of that cutoff. The limits of the relative errors in the MoSDeF study align with the central peak of the RR results, presumably because these simulations were implemented with a standardized procedure and the remaining errors are primarily statistical in nature. The RR work, which does not control for replicability via a centralized initialization procedure, thus has larger variance due to the human error stacking with the statistical error. Finally, the MoSDeF-Averaged distribution on the far right, with a height of 0.15%, gives an estimator of the expected closeness around the mean of the result given that 16 replicates are completed to find the solution. This 16 replicate average is a best practices approach to getting a high precision solution via well-controlled MoSDeF initiated simulation procedures and can be expected to yield a relative error of 0.2%; i.e., specific densities should have practical replicability to allow reporting of four significant figures.

**Figure 15:**
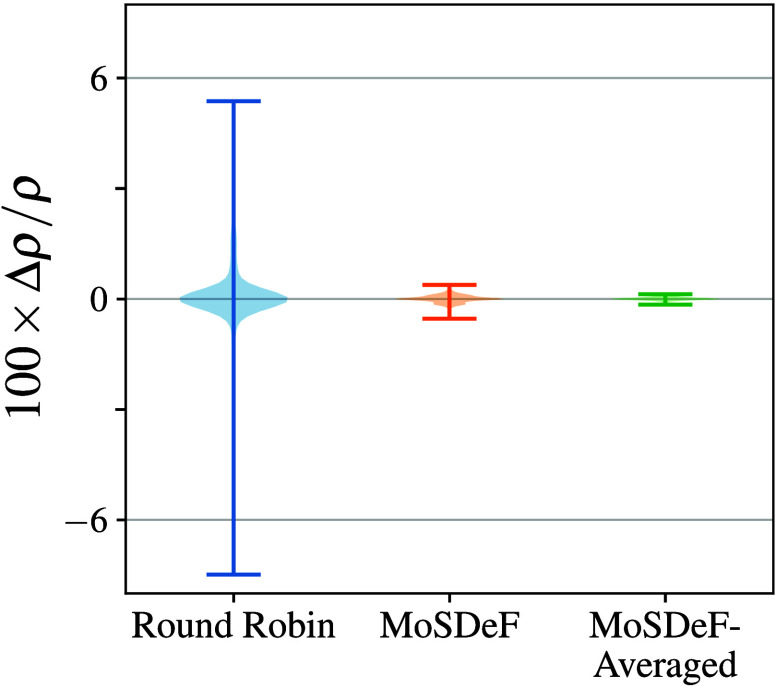
Round Robin paper total relative errors compared to MoSDeF simulation relative errors and MoSDeF engine by engine 16 replicate (best practice prediction) simulation errors.

## 5 Conclusions

This work was motivated by the reproducibility crisis in the MS community, highlighted by the thought-provoking results presented by Schappals et al.^35^ They highlighted an issue across the MS community: the expected level of replicability of even simple calculations performed without cross-code validation and well-defined simulation inputs across research groups and simulation engines can fall well outside the statistical variance. We believe we have provided a counterexample of this by comparing similar molecular density calculations across simulation engines via the standardized MS input utilizing MoSDeF and workflow controlled via signac. This level of standardization of inputs and force fields could be achieved without MoSDeF, but with considerable difficulty, thus emphasizing the utility of MoSDeF. From these results, we address the three questions raised in Section 1.

Issue 1 is to determine what should be considered practical replicability across simulation engines. Using MoSDeF, we have been able to eliminate many sources of error from the initialization, parametrization of molecular models, and writing syntactically correct molecular input files to different simulation engines. This reduction in error allows us to set a precedent for what can be considered practical replicability if the published force field is correctly applied and a reasonable implementation is chosen for the given software. We note that the scope of these differences is found in the context of unary systems for a set of simple molecular models (using pairwise additive nonbonded interactions), and more complex simulations would be expected to accumulate greater deviations. Errors found in the single-point energy contribute around 0.005%, which can be thought of as a lower bound for these values and is presumed to be due to small differences in input structure. Regardless of the exact boundary of MS practical replicability, which should be application specific with more complex systems requiring larger tolerances, the errors found in the RR study on the order of 7.5% can clearly be attributed to outliers generated through improper application of the force field or other gross implementation differences.

Issue 2 is an exploration of different error sources encountered in MS. The reduction in error sources via MoSDeF allows for identification and cataloging of the specific impacts of individual errors. We systematically add these differences back in across specific engines to understand the degrees of their effects on the density results. Differences in bond constraints, which result in a modified version of the OPLS-AA force field, result in error of 0.4% for ethanol–OPLS. Rounding differences of the LJ well depth of 0.06% result in density deviation of 0.02%, which has occurred in the version of the “SPC/E” force field that is packaged with LAMMPS, but it should be acknowledged that it has diverged from the original SPC/E force field.^48^ The implementation of long-range LJ correction is also explored, with the hard-cutoff, shifted LJ cutoff, and energy-pressure correction providing relative errors of 2%, in line with recent publication regarding TIP3P water, which showed 2.1% relative error without a shifted long-range correction.^87^ The effects of MD and MC treatments are found after high statistical power tests for TraPPE-UA methane. The density differences are found to be 0.02% and chiefly arise from the error in the MD approximation of force continuity at the cutoff radius. Errors that arise from changes to the force field or improper application of that force field can be considered “user error” in the MS process. Other systematic implementation differences studied or discussed herein are necessary for realistic MS, but they should be evaluated with caution, and care should be taken to document the choices made. Effects of time step and different ensemble controllers are well studied, but even more work needs to be done to explore the effects of these errors on other MS outputs.^88–90^

Issue 3 is related to the identification of best MS practices to reduce the systematic errors that can arise. An order of magnitude improvement was found by using a TRUE MS setup instead of trying to reproduce data strictly from written specifications. This is a clear indication that the MS community needs to adopt standardized inputs and publish the exact versions, source files, and scripted methods used to generate the MS inputs. Using a project manager, such as signac, will provide simulation templates and clearly indicate how the inputs are passed to each engine. Along these lines, users need to be careful of prepackaged force fields, as the process of transferring the parameters from the literature to these files is a documented source of error and results in incorrect MS. If possible, force field implementation can be validated across various engines by simply checking the single point energy of a single configuration. This identifies many such user differences discussed in Section 3.1 and is a highly recommended step, especially if using a tool that can output to multiple engines simultaneously, such as MoSDeF. Simulation software developers should test against benchmark standard values, such as those provided for simple molecules in this work, to catch bugs that may be introduced in later versions, or in the development of new simulation engines. While Schappals et al.^35^ emphasized software bugs as a large factor limiting practical replicability, the current study indicates very consistent data (always within the statistical uncertainties for all systems) among the three MC engines and for methane and water among the three MD engines. For methane, the difference between the MC and MD engines can be reduced by using a larger cutoff distance.

Further effort must be made to understand the impacts of force field application errors and implementation differences across more complex models. Additionally, improved communication and accessibility of code across simulation engines are useful tools for validation of methods as they stand in our software tools. In summary, as the MS community grows, it needs to set stronger standards for both the performance and reporting results. Development of novel and exciting methods will continue to compound the array of errors that can creep into simulations; therefore, it is imperative that these standards are set and adhered to such that inconsistencies, bugs, and faulty assumptions can be quickly diagnosed. Using such standards, practical replicability and even, in some cases, statistical equivalence can be achieved. Finally, it is worth noting that the consistency achieved between the various simulation engines and methodologies is attributable to the use of MoSDeF in ensuring force field consistency and the accuracy in exporting setup files for the different simulation engines. This study has provided extensive validation tests for the community-developed MS software.

## Supplementary Material



## Notes

Detailed instructions, including code, to reproduce the calculations reported in this paper. Tables of data derived from the various simulation engines. Graphs of kinetic energy distributions sampled by the MD engine thermostats. Details of simulation results for OPLS-AA Ethanol. Details of the comparison between MC and MD. Comparison of round robin results with this study. (PDF)
