# Supplementary material for: Achieving Reproducibility and Replicability of Molecular Dynamics and Monte Carlo Simulations Using the Molecular Simulation Design Framework (MoSDeF)
Source: J Chem Eng Data. 2025 May 27;70(6):2178–99. doi: 10.1021/acs.jced.5c00010 (PMC12169611; doi:10.1021/acs.jced.5c00010)
Supplement: Supplementary file 1 [file je5c00010_si_001.pdf]

Supporting Information: Achieving Reproducibility and Replicability of Molecular Dynamics and Monte Carlo Simulations Using the Molecular Simulation Design Framework (MoSDeF)

Nicholas C. Craven[1,12] Ramanish Singh[2,3,12] Co D. Quach[4,12] Justin B. Gilmer[1] Brad Crawford[5,6] Eliseo Marin-Rimoldi,[7] Ryan Smith,[7] Ryan DeFever,[7] Maxim S. Dyukov,[5] Jenny W. Fothergill[8] Chris Jones,[8] Timothy C. Moore,[9] Brandon L. Butler,[9] Joshua A. Anderson,[9] Christopher R. Iacovella,[4] Eric Jankowski[8] Edward J. Maginn,[7] Jeffrey J. Potoff[5] Sharon C. Glotzer,[9,10] Peter T. Cummings,[*,4,11] Clare M^c^Cabe,[*,4,11] and J. Ilja Siepmann[*,3,2]

[1]Interdisciplinary Materials Science Program, Vanderbilt University, Nashville, TN 37235-0106, USA
[2]Department of Chemical Engineering and Materials Science, University of Minnesota, Minneapolis, MN 55455-0132, USA
[3]Department of Chemistry and Chemical Theory Center, University of Minnesota, Minneapolis, MN 55455-0431, USA
[4]Department of Chemical and Biomolecular Engineering, Vanderbilt University, Nashville, TN 37235-1604, USA
[5]Department of Chemical Engineering, Wayne State University, Detroit, MI 48202-4050, USA
[6]Atomfold, PA 15478, USA
[7]Department of Chemical and Biomolecular Engineering, University of Notre Dame, Notre Dame, IN, USA
[8]Micron School of Materials Science and Engineering, Boise State University, Boise, ID 83725, USA
[9]Department of Chemical Engineering, University of Michigan, Ann Arbor, Michigan 48109-2800, USA
[10]Biointerfaces Institute, University of Michigan, Ann Arbor, Michigan 48109-2800, USA
[11]School of Engineering and Physical Sciences, Heriot-Watt University, Scotland EH14 4AS,United Kingdom
[12]N.C.C., R.S., and C.D.Q. contributed equally to this paper

* E-mail: p.cummings@hw.ac.uk; c.mccabe@hw.ac.uk; siepmann@umn.edu

S1 Reproducing The Study

In an effort to make our work reproducible and adhere to the TRUE principle, all steps to run and analyze simulations conducted in this study are included. Below are detailed instructions necessary to install the required software packages. For the packages that are installed from source code or through `pip`, we recommend installing them in a single directory on your machine. For this example, we will use the directory, `software`, as the location for the packages installed from the source code.

S1.1 Installation of required software

S1.1.1 Installation of the `mamba` Package Manager

We recommend using `mamba` package manager, due to its better performance compared to `Anaconda`, to host the Python environment utilized in this study. The `mamba` package manager can be installed by running the following commands in your shell session:

The $ denotes a line in your terminal emulator and is not part of the command.

For MacOS:

```
$ cd
$ curl micro.mamba.pm/install.sh | zsh
```

For GNU/Linux:

```
$ cd
$ curl micro.mamba.pm/install.sh | bash
```

A series of prompts will install the package based on your preferences. The default location, your home directory, can be expected to work. Please refer to the `mamba` documentation (`https://mamba.readthedocs.io/en/latest/`) for additional help. You may have to restart your current terminal to execute `mamba` commands. We note the `mamba` package manager will also accept `mamba` commands in place of `conda`, and the two commands can be used interchangeably.

S1.1.2 Creating the mamba Environment

Once mamba has been installed, the remaining software packages and libraries required to run and analyze the simulations can be installed follows the instructions described herein. Start by cloning the reproducibility_study repository and creating a new mamba environment with Python 3.8 as the base Python interpreter. A mamba environment is a directory that contains all of the packages and libraries based on your installation instructions. Multiple environments can be created on a single machine that contain different packages and/or different versions of the same packages. The environments are independent of each other, and modifications of one specific environment will not affect packages in other environments. Environments can easily be swapped by *deactivating* an environment and *activating* another environment. To simplify the process, an environment.yml file is included in the reproducibility_study repository. This file contains all the information required for mamba to install the required packages in the mosdef-study38 environment. Note that some packages cannot be installed via mamba, and instructions to install such packages are outlined below. All simulation and analysis steps should be run within the mosdef-study38 environment. All of the Python code necessary to initialize, run, and analyze the MC and MD simulations performed in this study are contained within the reproducibility_study repository on GitHub. Once the mosdef-study38 mamba environment has been created and activated, the reproducibility_study can be installed via pip. To create the mosdef-study38 environment and install the reproducibility_study execute the following commands:

```
# create and change into `software` directory
$ mkdir software
$ cd software
# git clone reproducibility_study
$ git clone https://github.com/mosdef-hub/reproducibility_study.git
$ cd reproducibility_study
# create and activate the `mosdef-study38` environment
$ mamba env create -f environment.yml
```

```
$ mamba activate mosdef-study38
# install the reproducibility_study
$ pip install -e .
```

Follow the series of command prompts to install all of the packages and their dependencies. A list of all packages installed in your current `mamba` environment can be viewed by running `$ mamba list`.

S1.1.3 Installation of MCCCS-MN

`MCCCS-MN` (`https://siepmann.chem.umn.edu/software`) is an open-source MC simulation software developed by the Siepmann research group at the University of Minnesota that can be used to simulate multi-component molecular systems in the NVT, NpT, μVT, $NVT-$Gibbs, and $NpT-$Gibbs ensembles. `MCCCS-MN` can be downloaded from this GitHub repository:
`https://github.com/ramanishsingh/MCCCS-MN-MoSDeF_ReproducibilityProject`. An older version can be downloaded from here:
`https://github.com/SiepmannGroup/PartialMolarProperties`. The software can be compiled using the following commands:

```
$ MCCCS-MN requires cmake and intel compilers for installation, which should be already
↪  be installed in this setup.
$ module load cmake
$ module load intel
# For cmake
$ mamba activate mosdef-study38
$ mamba install -c conda-forge cmake
#
# Cloning the repo in the home directory
$ cd software
$ git clone https://github.com/ramanishsingh/MCCCS-MN-MoSDeF_ReproducibilityProject
$ cd MCCCS-MN-MoSDeF_ReproducibilityProject/MCCCS-MN/
```

```
$ mkdir exe-mosdef; cd exe-mosdef
$ FC=ifort cmake -DDOUBLE_PRECISION=ON -DUSE_MPI=OFF -DUSE_OPENMP=OFF -DUSE_OWN=ON
↪  -DCMAKE_BUILD_TYPE=RELEASE
↪  software/MCCCS-MN-MoSDeF_ReproducibilityProject/MCCCS-MN/
# 1. assuming the code is in software/MCCCS-MN-MoSDeF_ReproducibilityProject/MCCCS-MN/
↪  directory
# 2. ccmake can be used in place of cmake for a graphical interface
$ make -j8
# MCCCS-MN is installed in
↪  software/MCCCS-MN-MoSDeF_ReproducibilityProject/MCCCS-MN/exe-mosdef/src directory
# It can be run using
↪  software/MCCCS-MN-MoSDeF_ReproducibilityProject/MCCCS-MN/exe-mosdef/src/topmon
```

S1.1.4 Installation of LAMMPS

LAMMPS was run using version 23Jun2022. The required tarball can be found here (https://download.lammps.org/tars/). In order to install on a cluster, follow these instructions for installing
(https://docs.lammps.org/Install_tarball.html#download-source-and-documentation-as-a-tar
and building (https://docs.lammps.org/Build.html).

S1.1.5 Installation of GOMC

GOMC (https://github.com/GOMC-WSU/GOMC.git) is one of the three MC engines utilized in this study. Installation of GOMC requires a working C/C++ compiler; please consult the user manual for detailed information: (https://gomc-wsu.github.io/Manual/software_requirements.html). The GOMC source code can be cloned from the GitHub repository: https://github.com/GOMC-WSU/GOMC.git. The instructions to install/compile GOMC are located in the GOMC Manual (https://gomc-wsu.github.io/Manual/compiling.html); however, for this project, some additional steps are required to determine the path of the compiled GOMC files to your bin directory, which are also specified below:

```
# GOMC requires cmake for compilation, which should already be installed in this setup.
$ module load cmake # if the cmake is available at your cluster
# However, if it is not, add it with the following commands via mamba:
$ mamba activate mosdef-study38
$ mamba install -c conda-forge cmake

# change into `software` directory
$ cd software
# clone GOMC
$ git clone https://github.com/GOMC-WSU/GOMC.git
$ cd GOMC
$ chmod u+x metamake.sh
$ ./metamake.sh
# once compiled, the executable should be located in the bin directory
$ ls ./bin

# Create a temporary and direct path to the GOMC binary files in the software/GOMC/bin
↪  directory.
# go to the GOMC directory
$ cd software/GOMC

# add the GOMC bin folder to our path, so we can find the executable no matter what the
↪  directory
# (i.e., find the GOMC_CPU_GCMC, GOMC_CPU_GEMC, GOMC_CPU_NVT, etc., files)
# note: the below would need to be completed every time you open a new terminal window.

# Note: these last two steps will need to be repeated if the terminal session is closed
# unless they are added to the shell configuration files (.bash_profile for bash or
↪  .zshrc for zsh).
# Create the temporary variable
$ LOC_GOMC="`pwd`/bin"

#Create a temporary GOMC bin directory path
```

```
$ export PATH="$PATH:$LOC_GOMC"
```

The section provided above is one method to access the compiled GOMC executables that were just created. The user will need to re-run the last 2 commands, which define and export the GOMC binary path to their shell environment, every time the terminal is reset. The gomc_binary_path variable in the GOMC project.py file will need to be set to "". The next code block provides an alternative way to access the GOMC executable's path if the user wants to reference and list the full path to the GOMC binaries, like was originally done in this work. To utilize this method, the user will need to replace the gomc_binary_path in the GOMC project.py with the full path to the GOMC binary directory on the respective computer or HPC, but will not need to define and export the executable path in their shell environment.

```
# obtaining the explicit path, "full_path_to_GOMC_bin_folder", to the GOMC executable
↪  using the 'pwd' command, assuming you are already in the GOMC directory.
$ cd software/GOMC/bin
$ pwd

# The examples below illustrate how to run a single GOMC job, but the instructions
↪  above with signac handle all of this automatically for the user.

# Example 1 (GCMC executable path with executable file):
↪  "full_path_to_GOMC_bin_folder"/GOMC_CPU_GCMC ;
# Example 2 (general GOMC executable path with executable file) :
↪  "full_path_to_GOMC_bin_folder"/GOMC_XXX_aaaa
# Example 3 (terminal code to run GOMC with Y cores from the directory containing the
↪  in.conf file) :  "full_path_to_GOMC_bin_folder"/GOMC_XXX_aaaa +pY in.conf > out.dat
```

The full path, listed in the terminal, plus the GOMC executable is can be used to run GOMC directly without setting up the GOMC PATH every time the user opens a new terminal window. Some examples are provided to show this method for directly running the GOMC software. Note: the in.conf is the existing configuration file, out.dat is the name of the output file,

and the items in quotation marks (`""`) are user-specific paths obtained by the entering `pwd` command in the above step (i.e., `"full_path_to_GOMC_bin_folder"`).

S1.1.6 Downloading workspace registry

The workspaces in each of the projects are downloadable and installable via Deep Blue Repositories. This can be accessed using the DOI https://doi.org/10.7302/fdqw-jy80, and instructions should be followed to access the data locally in your signac project. The instructions are available in the Data Repo/README.md file.

S1.2 Instructions to run simulations

Below are detailed instructions to run all molecular simulation and analysis steps contained with the `reproducibility_study`. Please note that small differences in the final results may exist due to different compute architectures, parallelization schemes, and random seeds used to run the simulations. Also note that analysis for `signac` projects may not successfully run until all jobs have been completed. We note, this instruction is tailored to run the main set of simulations, i.e., evaluation of densities of molecules via different simulation engines. The project also have subprojects, whose purposes were to investigate how different nuances in the system set up could affect the final results, e.g., effect of system size, effect of bonds treatment, etc. These subprojects share similar directories structure and can be carried in similar fashion/instruction, differed in only the location where the commands need to be executed. Each engine was responsible for a set of jobs created in the communal workspace directory and had a different control file (`project.py`), which specify sequences of operations that necessary to progress through the simulation workflows. Using the utilities provided by the `signac` and `signac-flow` libraries, these sequences have been organized with and post conditions of each steps, allowing for an semi-automated execution of the workflow. Due to the computing resources requires to carry out these simulations, it is highly advised to submit the simulation operations to a HPC system. The instructions

below will guide you to execute each workflow, iterating through all operations required to obtain the final results.

S1.2.1 Initialize the workspace

This project utilize the `signac` framework to manage the workspace and execute the workflow. Due to the shear amount of data generated in this study, we can only deliver a bare bone repository, containing on the codes necessary to reproduce the study. We note, for the majority of the project, `signac` v1.x was utilized. However, HOOMD-blue simulations were conducted at a later date to make use of the recently implemented Bussi thermostat in HOOMD-blue v4 and hence, utilized the more updated `signac` v2.x. To start the study, you will need to first initialize the workspace with signac following the commands below:

```
# activate the mosdef-study38 environment
$ mamba activate mosdef-study38
$ cd software/reproducibility_study/reproducibility_project/
$ python init.py
```

S1.2.2 Running LAMMPS Simulations

Available operations:

1. `build_lammps`
2. `lammps_cp_files`
3. `lammps_em_nvt`
4. `lammps_equil_npt`
5. `lammps_prod_npt`
6. `lammps_reformat_data`
7. `lammps_create_gsd`

```
# change into `reproducibility_study` directory
$ cd software/reproducibility_study/reproducibility_project/src/engines/lammps-VU
# Checking the status of all the jobs involved
$ python project.py status
# Submit jobs by operations
# The submission can be modified to include only certain amount of jobs to run (-n)
# as well as how many jobs to be bundled in one submission task
$ python project.py submit -o <operations> -n <n> --bundle=<bundle>
$ python project.py run
```

S1.2.3 Running HOOMD Simulations

Available operations:

1. `run_shrink`
2. `run_nvt`
3. `run_npt`
4. `check_equilibration_npt`
5. `check_equilibration_nvt`
6. `post_process`

As noted above, HOOMD-blue simulations were re-run at later time compared to the rest of the study to make use of the newly implemented features in HOOMD-blue v4, and utilized `signac` v2.x to control the workflow. Unfortunately, there are breaking changes introduced in the later version of `signac` and users will need to update the software to run HOOMD-Blue simulations. If the mosdef-study38 environment has been created according to the instruction above, only `hoomd` and `signac` version need to be updated, following the commands below:

```
$ mamba activate mosdef-study38
$ mamba update signac>=2
$ mamba update hoomd>=4
```

Alternatively, an additional `environment.yml` has also been provided with updated version specified:

```
$ cd software/reproducibility_study/reproducibility_project/src/engines/hoomd4
$ mamba env create -f environment.yml
$ mamba activate mosdef-study38
```

Then, users can continue the following instruction below to reproduce the HOOMD-blue simulations. Depends on your location on the terminal, the `cd` operation may looks different than the provided example.

```
# change into `reproducibility_study` directory
$ cd software/reproducibility_study/reproducibility_project/src/engines/hoomd4
# Checking the status of all the jobs involved
$ python project.py status
# Submit jobs by operations
# The submission can be modified to include only certain amount of jobs to run (-n)
# as well as how many jobs to be bundled in one submission task
$ python project.py submit -o <operations> -n <n> --bundle=<bundle>
$ python project.py run
```

S1.2.4 Running GROMACS Simulations

Available operations:

1. `init_job`
2. `gmx_em`
3. `gmx_nvt`
4. `gmx_npt_prod`

5. sample_npt_properties

6. gmx_nvt_prod

7. sample_nvt_properties

```
# change into `reproducibility_study` directory
$ cd software/reproducibility_study/reproducibility_project/src/engines/gromacs
# Checking the status of all the jobs involved
$ python project.py status
# Submit jobs by operations
# The submission can be modified to include only certain amount of jobs to run (-n)
# as well as how many jobs to be bundled in one submission task
$ python project.py submit -o <operations> -n <n> --bundle=<bundle>
$ python project.py run
```

S1.2.5 Running MCCCS-MN Simulations

MCCCS-MN simulations are also initialized, run, and managed with the signac framework. MCCCS-MN projects are contained within the reproducibility_project subdirectory in reproducibility_study directory. The following commands assume that the instructions for installing MCCCS-MN in previous section have been completed.

Available operations:

1. save_top

2. set_equil_replicates

3. set_prod_replicates

4. copy_files

5. copy_topmon

6. replace_keyword_fort_files_npt

7. `replace_keyword_fort_files_gemc`
8. `make_restart_file`
9. `run_melt`
10. `run_cool`
11. `run_equil`
12. `run_prod`
13. `convert_to_txt`
14. `convert_to_gsd`

```
# Assuming you are in the reproducibility_project subdirectory
$ cd software/reproducibility_study/reproducibility_project/src/engines/mcccs
$ vi src/engines/mcccs/project.py
# In line 37, change the path to MCCCS-MN executable
# In line 29, modify the signac template you want to use for this project
$ python src/engines/mcccs/project.py run #It will run the jobs in the terminal
$ python src/engines/mcccs/project.py submit #It will submit jobs to your cluster
```

Jobs will need to be submitted to the cluster multiple times after the completion of each simulation stage. After the simulations are complete (i.e. all stages of all jobs are finished), no new jobs will get submitted to the cluster.

S1.2.6 Running GOMC Simulations

Available operations:

1. `initial_parameters`
2. `build_psf_pdb_gomc_conf`

3. `run_melt_equilb_NVT_gomc_command`
4. `run_equib_ensemble_gomc_command`
5. `run_production_run_gomc_command`
6. `part_5a_individual_simulations_analysis`
7. `part_5b_avg_std_dev_of_replicates_analysis`

```
# change into `reproducibility_study` directory
$ cd software/reproducibility_study/reproducibility_project/src/engines/gomc
# Checking the status of all the jobs involved
$ python project.py status

# Submit avajobs by operations
# The submission can be modified to include only certain amount of jobs to run (-n)
# as well as how many jobs to be bundled in one submission task
$ python project.py submit -o <operations> -n <n> --bundle=<bundle>

# If you wish to run the operation directly without a scheduler
$ python project.py run
```

S1.2.7 Running Cassandra Simulations

Available operations:

1. `run_cassandra`
2. `statistics`
3. `process_output`

```
# change into `reproducibility_study` directory
$ cd software/reproducibility_study/reproducibility_project/src/engines/cassandra
# Checking the status of all the jobs involved
```

```
$ python project.py status
# Submit jobs by operations
# The submission can be modified to include only certain amount of jobs to run (-n)
# as well as how many jobs to be bundled in one submission task
$ python project.py submit -o <operations> -n <n> --bundle=<bundle>
$ python project.py run
```

S1.3 Data Analysis Routine

While the individual analysis are done within the `project.py` of each individual engines. Once all simulations have beend done, we can calculate the aggregate statistics, averaging the properties from all simulations, grouping by the state points and molecule. The aggregate analysis procedure can be carried out as follow:

```
# change into `reproducibility_study` directory and initialize the aggregate_summary
↪  workspace
$ cd software/reproducibility_study/reproducibility_project/aggregate_summary
$ python aggregate_init.py
# Perform aggregate data analysis
$ python project-analysis.py run
# Checking the status of all the jobs involved
$ python project.py status
# Submit jobs by operations
# The submission can be modified to include only certain amount of jobs to run (-n)
# as well as how many jobs to be bundled in one submission task
$ python project.py run
```

S1.4 Reproducing of Subprojects

The project also includes several subprojects, in which we attempted to determine the impacts of different factors, such as system size and bond treatment, on the final simulation results. These subprojects have the same directory structure as the main project and can be

carried out using the same instructions listed above, differed only by the location to execute the control project.py.

S1.5 Removing the `mamba` Environment

If you are finished running the simulations and would like to remove the `mamba` environment and all relevant files, the following command can be run:

```
# Remove `mosdef-study38` environment
$ mamba remove --name mosdef-study38 --all
```

S2 Tables

S2.1 Molecular Weights

Table S1: Molecular weight (MW [g/mol]) of each bead (atom for all-atom models) from the force field files used for the simulations. Refer to Figure 1 for bead nomenclature.

Molecule	Bead	MW
Methane-TraPPE	CH_4	16.0430
Pentane-TraPPE	CH_3	15.0350
Pentane-TraPPE	CH_2	14.0270
Benzene-TraPPE	CH	13.0190
Water-SPC/E	O	15.9994
Water-SPC/E	H	1.0080
Ethanol-OPLS	C	12.0110
Ethanol-OPLS	O	15.9994
Ethanol-OPLS	H	1.0080

S2.2 Engine Units

Table S2: Native length and energy units for the simulation engines used

Simulation engine	Length	Energy	Notes
`LAMMPS` (MD)	Å	kcal/mol	LAMMPS real units
`GROMACS` (MD)	nm	kJ/mol	
`HOOMD-blue` (MD)	unitless	unitless	Self-consistent unit systems,<br>reference length: 1 nm, reference energy: 1 kJ
`MCCCS-MN` (MC)	Å	K	
`GOMC` (MC)	Å	kcal/mol	Output in Å and K
`Cassandra` (MC)	Å	K	Different units (kJ/mol) for<br>OPLS and CHARMM dihedrals

S2.3 Packmol Initial Configurations

Table S3: Bond lengths for initial configurations of each liquid molecule box set to force field bond lengths by Packmol

Molecule	Bonded Atoms	Force Field Parameter Length nm	Mean Bond Length nm	Standard Deviation of Bond Length nm
PentaneUA	CH2-CH3	0.1540	0.1540000144	4.44E-08
PentaneUA	CH2-CH2	0.1540	0.1539999912	4.06E-08
BenzeneUA	CH-CH	0.1400	0.1399999672	3.57E-07
WaterSPCE	OW-HW	0.1000	0.0999999892	4.24E-08
EthanolAA	C-C	0.1529	0.1528995123	4.23E-08
EthanolAA	C-O	0.1410	0.1410006150	4.01E-08
EthanolAA	O-H	0.0945	0.0944999999	4.16E-08

S2.4 Single Point Energy Tables

A library of system snapshots was generated to ensure uniformity across all simulation engines for single-point energy calculations. Molecules were randomly positioned within simulation boxes using PACKMOL. Box sizes were determined based on system density data from literature sources. For each snapshot, single point energy was calculated using all the simulation engines used in this study.

Table S4: Single point energy for methane-TraPPE via different simulation engines

Simulation engine	Potential energy [kJ]	Total VdW energy [kJ]	Long range correction [kJ]
`LAMMPS` (MD)	536 743.5538	536 743.5538	−128.2312
`GROMACS` (MD)	536 736.4375	536 736.4427	−128.2448
`HOOMD-blue` (MD)	536 736.4702	536 736.4702	−128.2295
`MCCCS-MN` (MC)	536 736.6741	536 736.6741	−128.2296
`GOMC` (MC)	536 736.5544	536 736.5538	−128.2296
`Cassandra` (MC)	536 737.2800	536 737.2803	−128.2297

Table S5: Single point energy for pentane-TraPPE via different simulation engines

Simulation engine	Potential energy [kJ]	Total VdW energy [kJ]	Long range correction [kJ]	Angles energy [kJ]	Dihedrals energy [kJ]
`LAMMPS` (MD)	537 739.1201	537 739.1201	−181.1081	6.2630×10^{-8}	2.9225×10^{-9}
`GROMACS` (MD)	537 737.1875	537 737.1640	−181.1485	6.8130×10^{-6}	0.0057
`HOOMD-blue` (MD)	537 737.4840	537 737.4840	−181.1078	6.6862×10^{-6}	2.9219×10^{-9}
`MCCCS-MN` (MC)	537 737.7928	537 737.7928	−181.1079	6.2630×10^{-8}	2.9217×10^{-9}
`GOMC` (MC)	537 737.3920	537 737.3815	−181.1078	7.0136×10^{-6}	0.0100
`Cassandra` (MC)	537 738.4000	537 738.4014	−181.1081	6.3703×10^{-8}	2.9359×10^{-9}

Table S6: Single point energy for rigid benzene-TraPPE via different simulation engines

Simulation engine	Potential energy [kJ]	Total VdW energy [kJ]	Long range correction [kJ]
`LAMMPS` (MD)	388 924.2236	388 924.2236	−250.5492
`GROMACS` (MD)	388 939.1250	388 939.1318	−250.5869
`HOOMD-blue` (MD)	388 939.0095	388 939.0095	−250.5587
`MCCCS-MN` (MC)	388 939.0096	388 939.0095	−250.5587
`GOMC` (MC)	388 939.0511	388 939.0512	−250.5587
`Cassandra` (MC)	388 939.8200	388 939.8210	−250.5590

Table S7: Single point energy for water-SPC/E via different simulation engines

Simulation engine	Potential energy [kJ]	Total VdW energy [kJ]	Long range correction [kJ]	Total electrostatics [kJ]
LAMMPS (MD)	57 176.3853	76 663.7135	−275.6510	−19 487.3282
GROMACS (MD)	57 175.9180	76 663.5498	−275.8252	−19 487.6299
HOOMD-blue (MD)	57 176.1610	76 663.7607	−275.6512	−19 487.5996
MCCCS-MN (MC)	57 175.4228	76 664.1983	−275.6528	−19 488.7755
GOMC (MC)	57 175.4333	76 663.7672	−275.6512	−19 488.3339
Cassandra (MC)	57 175.7780	76 663.8435	−275.6515	−19 488.0703

Table S8: Single point energy for ethanol-OPLS via different simulation engines

Simulation engine	Potential energy [kJ]	Total VdW energy [kJ]	Tail energy [kJ]	Total electro-statics [kJ]	Bonds energy [kJ]	Angles energy [kJ]	Dihedrals energy [kJ]
`LAMMPS` (MD)	31 409.5039	19 408.2707	−417.8758	3575.3313	5.9652×10^{-5}	7233.2705	1192.6314
`GROMACS` (MD)	31 409.1484	19 408.1896	−417.9560	3575.0720	6.2869×10^{-5}	7233.2656	1192.6206
`HOOMD-blue` (MD)	31 409.6390	19 408.2707	−417.8758	3575.4672	5.9652×10^{-5}	7233.2705	1192.6305
`MCCCS-MN` (MC)	31 409.5198	19 408.4054	−417.8786	3575.1597	0.0	7233.3163	1192.6385
`GOMC` (MC)	31 409.5604	22 407.8831	−417.8758	575.7854	6.2253×10^{-5}	7233.2714	1192.6205
`Cassandra` (MC)	31 409.4690	19 408.1143	−417.8762	3575.3416	N/R	7233.2478	1192.7656

We note, for GOMC engine, the short range electrostatics energy was reported together with short range LJ. The value was then added into the"Total VdW energy" and not "Total electrostatics", which explained their differences with the rest of the engines studied as seen Table S8.

S2.5 Density tables

This subsection contains the densities, ρ, and percent relative density deviations (as shown in Equation 1, Section 2.4 in the main text), δ, data tables for the systems studied.

Table S9: Densities, ρ, and percent relative density deviations, δ, and their confidence intervals, u, for TraPPE molecules calculated via different simulation engines

Molecule	State point	Simulation engine	ρ [kg/m^3] value	ρ u	δ [unitless] value	δ u
Methane-TraPPE	140 K, 1318 kPa	LAMMPS (MD)	375.961	0.024	0.0276	0.0065
		GROMACS (MD)	375.957	0.030	0.0265	0.0080
		HOOMD-blue (MD)	375.955	0.019	0.0260	0.0051
		MCCCS-MN (MC)	375.76	0.10	-0.026	0.027
		GOMC (MC)	375.789	0.064	-0.018	0.017
		Cassandra (MC)	375.724	0.096	-0.036	0.026
Pentane-TraPPE	372.0 K, 1402 kPa	GROMACS (MD)	541.91	0.16	0.106	0.030
		HOOMD-blue (MD)	541.639	0.076	0.056	0.014
		MCCCS-MN (MC)	541.09	0.19	-0.046	0.035
		GOMC (MC)	540.85	0.28	-0.090	0.052
		Cassandra (MC)	541.19	0.47	-0.027	0.086
Benzene-TraPPE	450 K, 2260 kPa	LAMMPS (MD)	697.67	0.13	-0.105	0.018
		HOOMD-blue (MD)	699.155	0.054	0.1084	0.0077
		MCCCS-MN (MC)	698.04	0.51	-0.051	0.074
		GOMC (MC)	698.69	0.45	0.042	0.064
		Cassandra (MC)	698.44	0.53	0.006	0.076

Table S10: Densities, ρ, and percent relative density deviations, δ, and their confidence intervals, u, for water-SPC/E calculated via different simulation engines

Molecule	State point	Simulation engine	ρ [kg/m^3]		δ [unitless]	
			value	u	value	u
Water-SPC/E	280 K, 101.325 kPa	`LAMMPS` (MD)	1006.87	0.11	0.008	0.011
		`GROMACS` (MD)	1006.732	0.079	-0.0054	0.0078
		`HOOMD-blue` (MD)	1006.616	0.048	-0.0169	0.0047
		`MCCCS-MN` (MC)	1007.05	0.76	0.027	0.075
		`GOMC` (MC)	1006.87	0.90	0.009	0.090
		`Cassandra` (MC)	1006.57	0.91	-0.021	0.090
	300 K, 101.325 kPa	`LAMMPS` (MD)	998.034	0.085	0.0194	0.0085
		`GROMACS` (MD)	997.869	0.055	0.0028	0.0055
		`HOOMD-blue` (MD)	997.784	0.038	-0.0057	0.0038
		`MCCCS-MN` (MC)	997.71	0.82	-0.013	0.082
		`GOMC` (MC)	997.89	0.65	0.005	0.066
		`Cassandra` (MC)	997.76	0.85	-0.008	0.086
	320 K, 101.325 kPa	`LAMMPS` (MD)	986.319	0.085	-0.0024	0.0087
		`GROMACS` (MD)	986.364	0.049	0.0021	0.0050
		`HOOMD-blue` (MD)	986.201	0.034	-0.0144	0.0035
		`MCCCS-MN` (MC)	986.14	0.49	-0.021	0.050
		`GOMC` (MC)	986.20	0.45	-0.015	0.046
		`Cassandra` (MC)	986.84	0.49	0.050	0.050

Table S11: Densities, ρ, and percent relative density deviations, δ, and their confidence intervals, u, for ethanol-OPLS calculated via different simulation engines

Molecule	State point	Simulation engine	ρ [kg/m^3] value	ρ u	δ [unitless] value	δ u
Ethanol-OPLS	280 K, 101.325 kPa	`LAMMPS` (MD)	815.94	0.16	0.186	0.019
		`GROMACS` (MD)	816.78	0.12	0.288	0.015
		`HOOMD-blue` (MD)	814.501	0.059	0.0089	0.0072
		`MCCCS-MN` (MC)	812.73	0.54	-0.208	0.066
		`GOMC` (MC)	813.13	0.77	-0.159	0.095
		`Cassandra` (MC)	813.49	0.73	-0.115	0.090
	300 K, 101.325 kPa	`LAMMPS` (MD)	795.460	0.096	0.293	0.012
		`GROMACS` (MD)	795.62	0.20	0.313	0.025
		`HOOMD-blue` (MD)	793.700	0.063	0.0707	0.0080
		`MCCCS-MN` (MC)	791.42	0.46	-0.217	0.058
		`GOMC` (MC)	791.35	0.73	-0.226	0.092
		`Cassandra` (MC)	791.28	0.48	-0.234	0.061
	320.0 K, 101.325 kPa	`LAMMPS` (MD)	773.89	0.15	0.400	0.020
		`GROMACS` (MD)	773.67	0.21	0.372	0.027
		`HOOMD-blue` (MD)	772.135	0.057	0.1729	0.0074
		`MCCCS-MN` (MC)	768.39	0.45	-0.313	0.059
		`GOMC` (MC)	768.01	0.64	-0.362	0.083
		`Cassandra` (MC)	768.72	0.46	-0.270	0.060

S2.6 Ethanol Bonds Conditions

Table S12: Densities, ρ, and percent relative density deviations, δ, and their confidence intervals, u, for Ethanol-OPLS calculated via different simulation engines under different bond conditions

Molecule	State point	Simulation Engine	Bond condition	ρ [kg/m^3] value	ρ u	δ [unitless] value	δ u
Ethanol-OPLS	280.0 K, 101.325 kPa	`LAMMPS` (MD)	Flexible	815.9	0.15	0.24	0.02
			Fixed OH	812.45	0.09	-0.19	0.01
		`MCCCS-MN` (MC)	Flexible	814.7	0.5	0.08	0.06
			Fixed	813.0	0.4	-0.13	0.05
	300 K, 101.325 kPa	`LAMMPS` (MD)	Flexible	795.46	0.09	0.33	0.01
			Fixed OH	791.2	0.1	-0.21	0.02
		`MCCCS-MN` (MC)	Flexible	793.2	0.3	0.05	0.04
			Fixed	791.4	0.2	-0.18	0.03
	320 K, 101.325 kPa	`LAMMPS` (MD)	Flexible	773.9	0.1	0.42	0.02
			Fixed OH	768.7	0.1	-0.25	0.02
		`MCCCS-MN` (MC)	Flexible	771.1	0.4	0.07	0.05
			Fixed	768.7	0.3	-0.24	0.04

S3 MD Kinetic Energies

The kinetic energy distributions sampled by the MD engine thermostats were tested to validate that they are approximately normal when sampled from the Boltzmann distribution. The following figures and p-values test this hypothesis for both the mean of the distribution and the variance. This testing was done via the `physical validation` python package and the code is located in the `src` directory of the Git repository.[1]

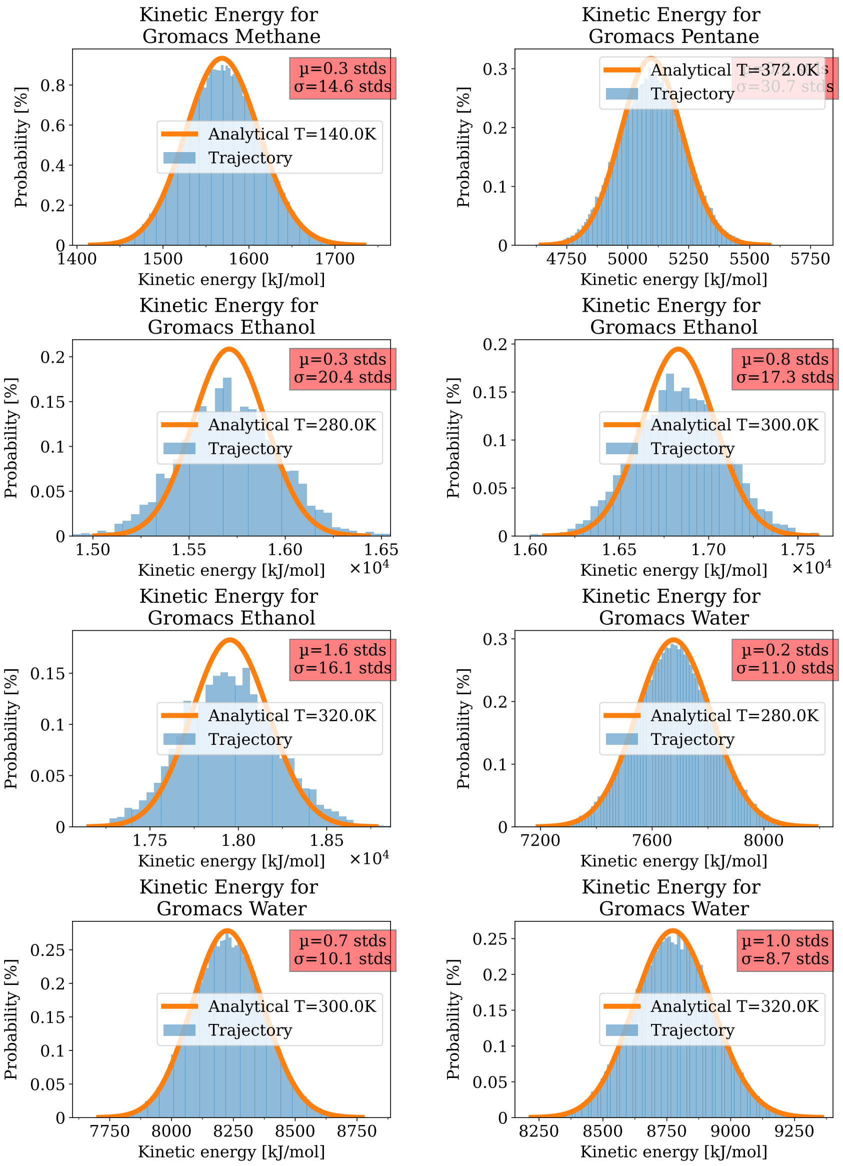

Figure S1: Kinetic energy distributions from equilibrated samples of GROMACS production phase for the molecules in this study. μ is the number of standard deviations the mean is from the analytical kinetic energy distribution, and σ is the same for the standard deviation difference from that analytical distribution.

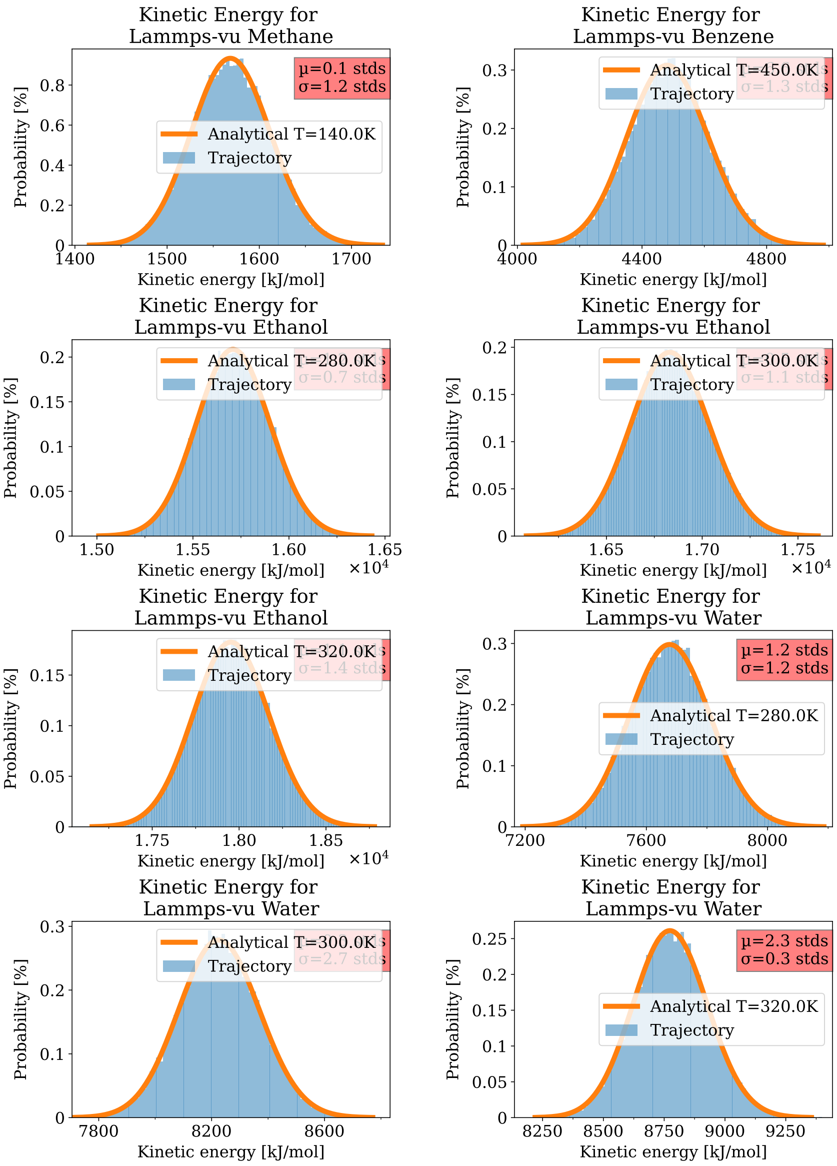

Figure S2: Kinetic energy distributions from equilibrated samples of LAMMPS production phase for the molecules in this study. μ is the number of standard deviations the mean is from the analytical kinetic energy distribution, and σ is the same for the standard deviation difference from that analytical distribution.

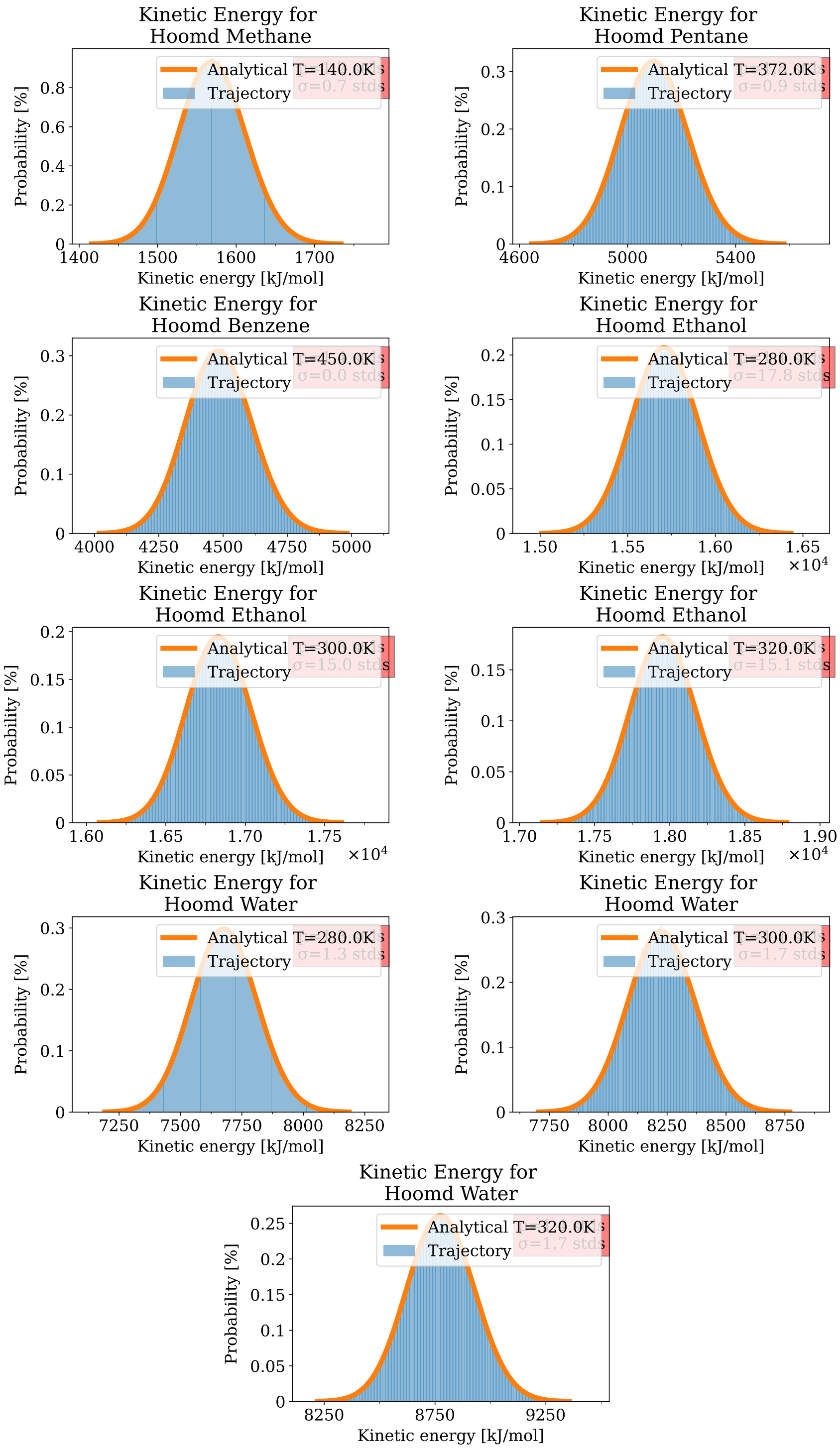

Figure S3: Kinetic energy distributions from equilibrated samples of `HOOMD-blue` production phase for the molecules in this study. μ is the number of standard deviations the mean is from the analytical kinetic energy distribution, and σ is the same for the standard deviation difference from that analytical distribution.

S4 OPLS-AA Ethanol

Figure S4, S5, S6 show the bond length distributions for the C-C, C-O, O-H, and C-H bonds for the MCCCS-MN-flex simulations at 280 K, 300 K, and 320 K, respectively. All bonds, except the O-H bonds, fluctuate around their equilibrium lengths. A noticeable elongation of around 2% in the mean length of the O-H bonds is observed in the flexible O-H bond simulations. Figures S7 and S8 show the RDF and CDF for the `MCCCS-MN` simulations, respectively. The CDF for flexible ethanol simulations is higher than that of fixed-bond ethanol simulations. These analyses suggest that ethanol molecules are packed more compactly in the flexible ethanol simulations as compared to fixed-bond ethanol simulations. The flexibility of the O-H bond enables the ethanol molecules to make more hydrogen bonds per molecule and pack more efficiently.

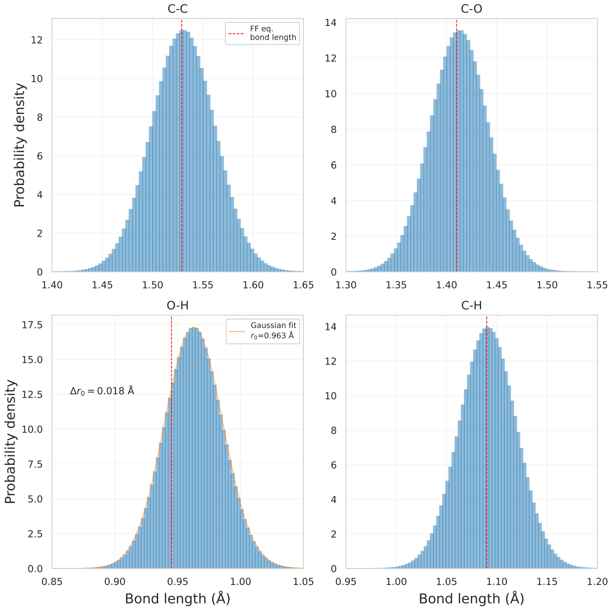

Figure S4: C-C (top left), C-O (top right), O-H (bottom left), and C-H (bottom right) bond length distribution at 280 K and 101.325 kPa for ethanol-OPLS from MCCCS-MN-flex simulations. Vertical red-dashed line shows the equilibrium bond length from OPLS–AA[2] forcefield. The O-H bond distribution is fit to a Gaussian function (orange) and Δr_0 denotes the difference between the Gaussian fit mean and FF equilibrium bond length.

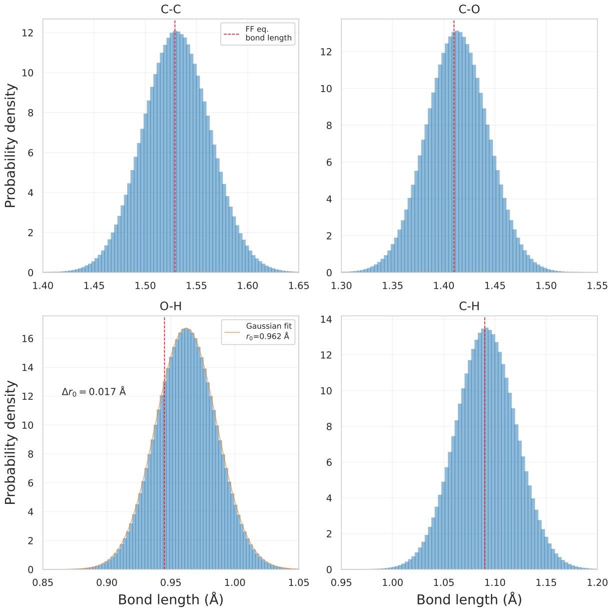

Figure S5: C-C (top left), C-O (top right), O-H (bottom left), and C-H (bottom right) bond length distribution at 300 K and 101.325 kPa for ethanol-OPLS from MCCCS-MN-flex simulations. Vertical red-dashed line shows the equilibrium bond length from OPLS–AA forcefield. The O-H bond distribution is fit to a Gaussian function (orange) and Δr_0 denotes the difference between the Gaussian fit mean and FF equilibrium bond length.

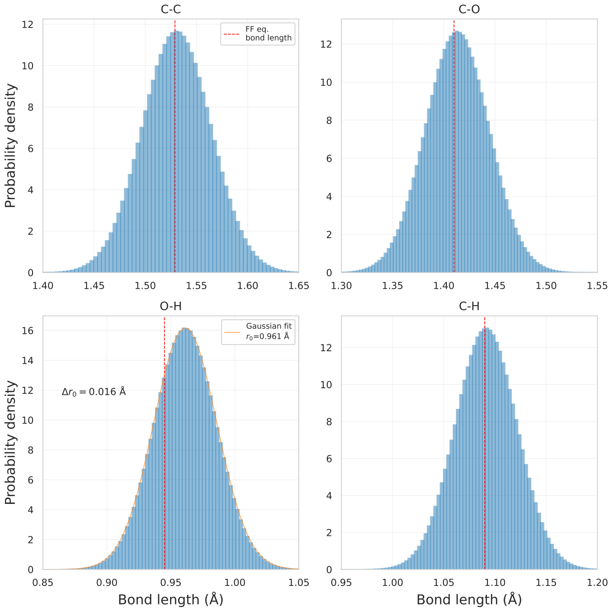

Figure S6: C-C (top left), C-O (top right), O-H (bottom left), and C-H (bottom right) bond length distribution at 320 K and 101.325 kPa for ethanol-OPLS from MCCCS-MN-flex simulations. Vertical red-dashed line shows the equilibrium bond length from OPLS–AA forcefield. The O-H bond distribution is fit to a Gaussian function (orange) and Δr_0 denotes the difference between the Gaussian fit mean and FF equilibrium bond length.

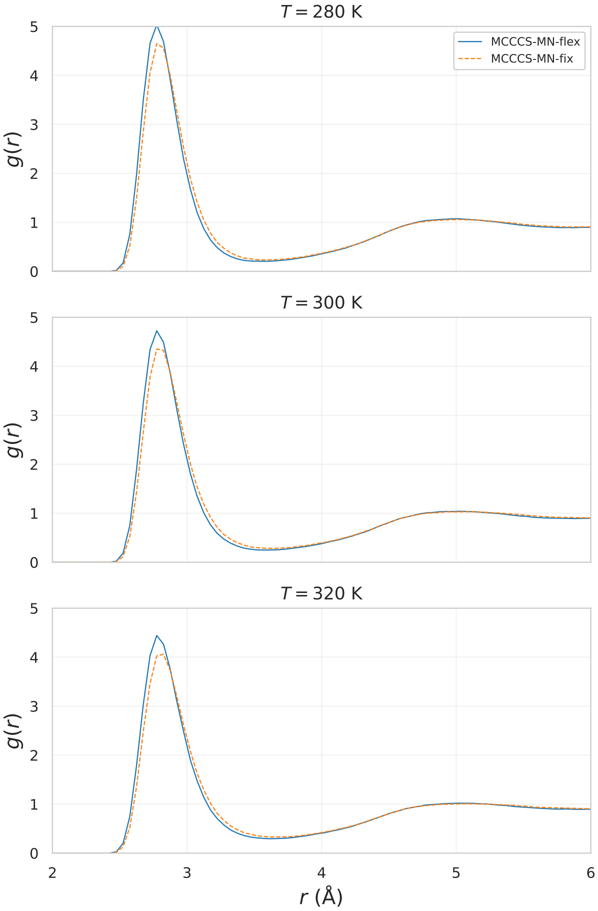

Figure S7: Radial distribution function ($g(r)$) for ethanol-OPLS MCCCS-MN-fix (blue) and MCCCS-MN-flex (dashed-orange) simulations at 280 K (top), 300 K (middle), and 320 K (bottom).

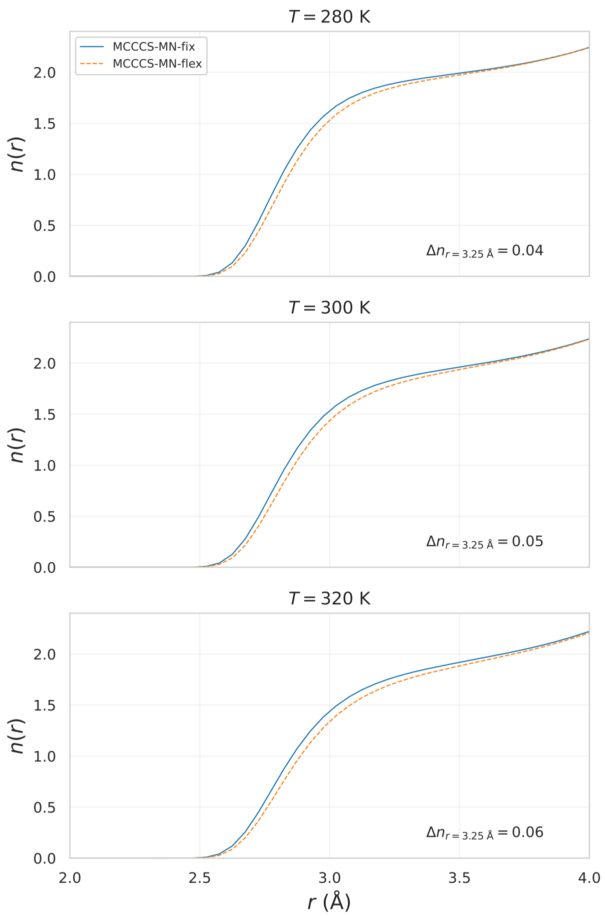

Figure S8: Number integral ($n(r)$) for ethanol-OPLS MCCCS-MN-fix (blue) and MCCCS-MN-flex (dashed-orange) simulations at 280 K (top), 300 K (middle), and 320 K (bottom). $\Delta n_{\text{r}=3.25\ \text{Å}}$ denotes the difference between MCCCS-MN-flex and MCCCS-MN-fix $n(r)$ values at $r = 3.25$ Å, i.e. the hydrogen bond distance cutoff.

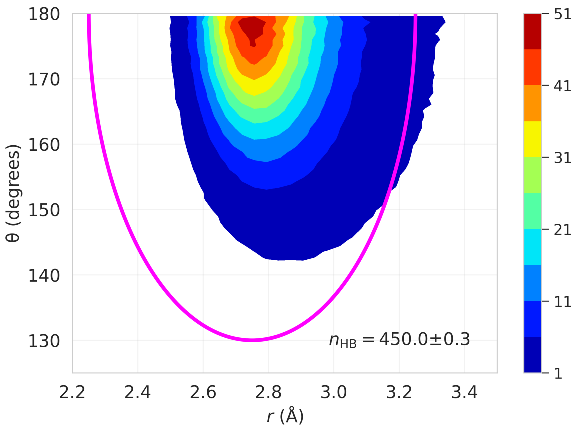

Figure S9: Radial-angular distribution function (RADF) for ethanol-OPLS MCCCS-MN-flex simulations at 280 K and 101.325 kPa. θ is the $\mathrm{O}--\mathrm{H}-\mathrm{O}$ angle and r is the $\mathrm{O}-\mathrm{O}$ distance. Hydrogen bond elliptical criterion boundary is shown in magenta, and the points (ethanol-ethanol pairs) lying inside the boundary are considered to be hydrogen bonded. n_{HB} denotes the number of hydrogen bonds.

S4.1 Long Range Correction Tables

Table S13: Densities, ρ, and percent relative density deviations, δ, and their confidence intervals, u, for methane-TraPPE calculated via different simulation engines using different long range treatments

Molecule	Long range treatment	Simulation engine	ρ [kg/m^3]		δ [unitless]	
			value	u	value	u
Methane-TraPPE	Hard cutoff	`LAMMPS` (MD)	363.20	0.06	-0.88	0.02
		`GROMACS` (MD)	363.22	0.04	-0.87	0.01
		`HOOMD-blue` (MD)	363.09	0.04	-0.91	0.01
		`MCCCS-MN` (MC)	369.64	0.08	0.88	0.02
		`GOMC` (MC)	369.66	0.07	0.89	0.02
		`Cassandra` (MC)	369.6	0.1	0.88	0.03
	Hard cutoff, with energy and pressure correction	`LAMMPS` (MD)	375.97	0.03	0.035	0.008
		`GROMACS` (MD)	375.96	0.04	0.03	0.01
		`HOOMD-blue` (MD)	375.90	0.03	0.016	0.007
		`MCCCS-MN` (MC)	375.7	0.1	-0.04	0.03
		`GOMC` (MC)	375.79	0.06	-0.01	0.02
		`Cassandra` (MC)	375.7	0.1	-0.03	0.02
	Shift cutoff	`LAMMPS` (MD)	363.2	0.1	0.02	0.02
		`GROMACS` (MD)	363.21	0.03	0.02	0.01
		`HOOMD-blue` (MD)	363.09	0.03	-0.010	0.009
		`MCCCS-MN` (MC)	363.04	0.07	-0.02	0.02
		`GOMC` (MC)	363.1	0.1	-0.01	0.03
		`Cassandra` (MC)	363.13	0.09	0.00	0.03

Table S14: Densities, ρ, and percent relative density deviations, δ, and their confidence intervals, u, for water-SPC/E calculated via different simulation engines using different long range treatments

Molecule	Long range treatment	Simulation engine	ρ [kg/m^3]		δ [unitless]	
			value	u	value	u
Water-SPC/E	Hard cutoff	`LAMMPS` (MD)	985.2	0.1	-0.30	0.01
		`GROMACS` (MD)	985.21	0.06	-0.304	0.006
		`HOOMD-blue` (MD)	985.05	0.07	-0.320	0.007
		`MCCCS-MN` (MC)	991.2	0.9	0.30	0.09
		`GOMC` (MC)	991.1	0.6	0.29	0.06
		`Cassandra` (MC)	991.5	1.0	0.3	0.1
	Hard cutoff, with energy and pressure correction	`LAMMPS` (MD)	998.0	0.1	0.02	0.01
		`GROMACS` (MD)	997.95	0.07	0.016	0.007
		`HOOMD-blue` (MD)	997.70	0.07	-0.008	0.007
		`MCCCS-MN` (MC)	997.9	0.6	0.01	0.07
		`GOMC` (MC)	997.9	0.6	0.01	0.06
		`Cassandra` (MC)	997.3	0.9	-0.05	0.09
	Shift cutoff	`LAMMPS` (MD)	985.4	0.1	0.02	0.01
		`GROMACS` (MD)	985.22	0.06	0.003	0.006
		`HOOMD-blue` (MD)	985.03	0.06	-0.016	0.006
		`MCCCS-MN` (MC)	985.2	0.7	0.00	0.07
		`GOMC` (MC)	985.0	0.6	-0.02	0.06
		`Cassandra` (MC)	985.2	0.7	0.00	0.07

S5 Comparing MC and MD

S5.1 Simulating a system of WCA particles

We performed simulations on a system of $N = 1728$ particles interacting via the Weeks-Chandler-Anderson (WCA) potential[3] at $p = 11\epsilon/\sigma^3$, $kT = 1\epsilon$ with `HOOMD-blue` (4.0) and `MCCCS-MN` with increasing timesteps. Both the WCA energy *and force* are 0 at the cutoff radius which removes the long range pressure correction from consideration and decreases the error due to discontinuous potentials compared to Lennard-Jones with a hard cutoff. Note that while the WCA force is continuous, *its derivatives are not.* We run MD simulations in `HOOMD-blue` with the Bussi thermostat[4] and the MTTK barostat[5] (with $\tau = 3\sigma\sqrt{m/\epsilon}$) for

$4 \cdot 10^6$ time steps with 32 independent replicates. We compare those to constant pressure MC simulations with `HOOMD-blue`[6] ($4 \cdot 10^6$ steps with one volume move and approximately N trial moves per step, 32 replicates) and `MCCCS-MN` ($80 \cdot 10^3$ Monte Carlo cycles to equilibrate and 120 $\cdot 10^3$ MC cycles for production with a volume move probability of = 0.0011, 16 replicates). The comparison presented in Table S15 demonstrates that MD simulations can yield average number densities consistent with MC simulations. Furthermore, the MC results obtained from both `MCCCS-MN` and `HOOMD-blue` exhibit statistical similarity.

Table S15: Number density, ρ, and its confidence interval, u, obtained for WCA simulations using `MCCCS-MN` and `HOOMD-blue`

Simulation engine	$\delta t/\sigma\sqrt{m/\epsilon}$	ρ/σ^{-3}	
		value	u
`MCCCS-MN` (MC)		0.91939	0.00004
`HOOMD-blue` (MC)		0.91941	0.00002
`HOOMD-blue` (MD)	0.0001	0.91940	0.00002
`HOOMD-blue` (MD)	0.0015	0.91939	0.00002
`HOOMD-blue` (MD)	0.0050	0.91912	0.00002

S5.2 Effect of time step on methane density from MD

As mentioned in the main text, the selection of a time step can have a significant impact on the outcomes of MD simulations. In our computational study, we employed a time step of 2 fs (`LAMMPS`) for the methane simulations. To ensure that this particular time step choice does not contribute to any discrepancies observed between the MC and MD results, we also performed additional simulations using a smaller time step of 0.5 fs. The density values obtained from these simulations are presented in Table S16. Notably, the statistical analysis reveals that the results obtained from both time step values exhibit no significant differences. Hence, it can be concluded that a time step of 2 fs for methane simulations is appropriate for our purposes.

Table S16: Methane density, ρ, and its confidence interval, u, calculated (900 molecule system, 256 independent simulations) using two different time step values in `LAMMPS`

Simulation engine	Time step [fs]	ρ [kg/m^3]	
		value	u
`LAMMPS` (MD)	2	375.93	0.02
`LAMMPS` (MD)	0.5	375.96	0.01

S6 RR to MoSDeF Comparisons

Figures here are alternative visualizations to the figures in Section 4 of the main text of the extents of error for this work compared to the RR simulations. They are shown as scatter plots of the raw relative deviations from all of the simulations for the RR work and MoSDeF work for each model studied. The results are compared for comparisons across all engines (Figure 11 and 13) and when treating the MC and MD engines as separate groupings for which to compare errors to the means (Figure 12 and 14).

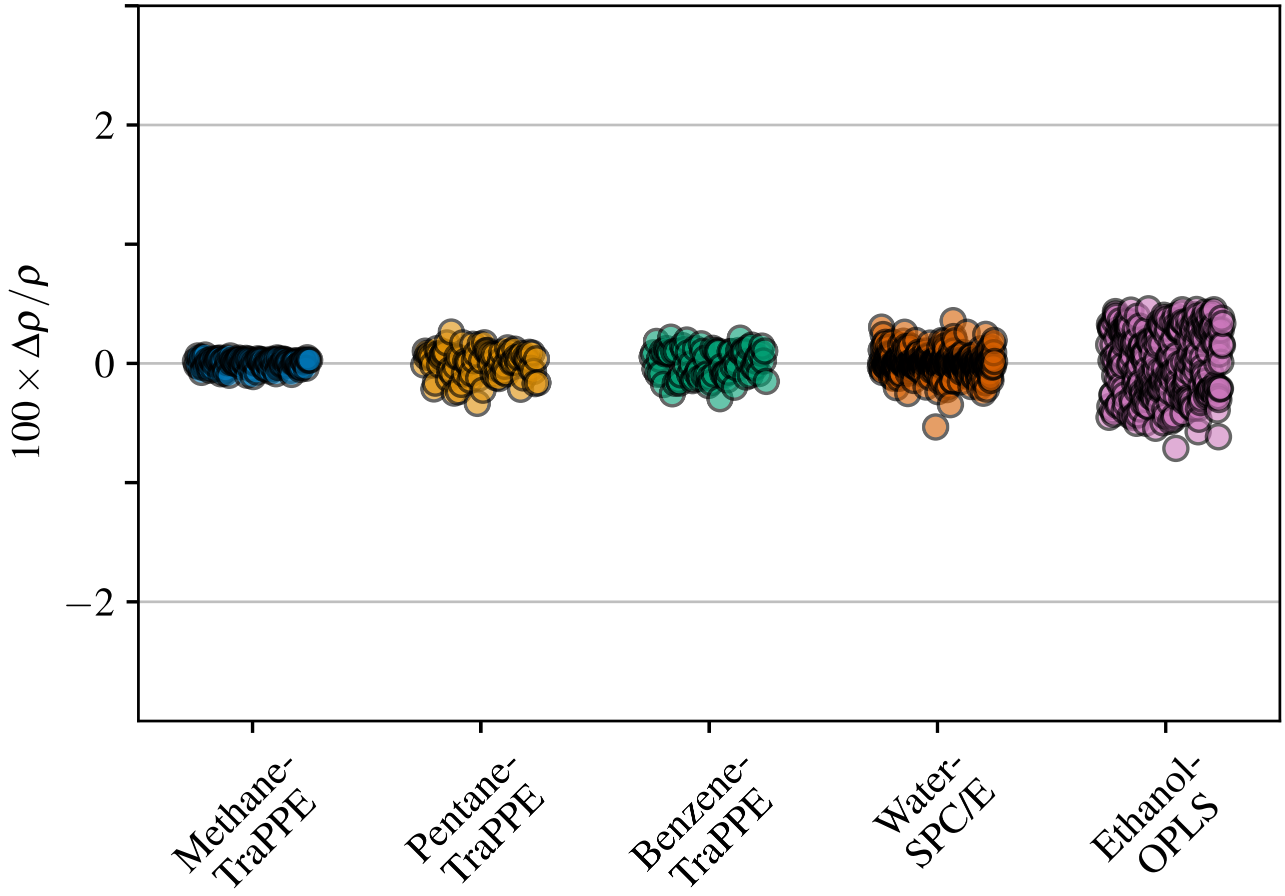

Figure S10: Simulation relative deviation for MoSDeF models-force fields when grouping MC or MD engines together (as opposed to Figure 12).

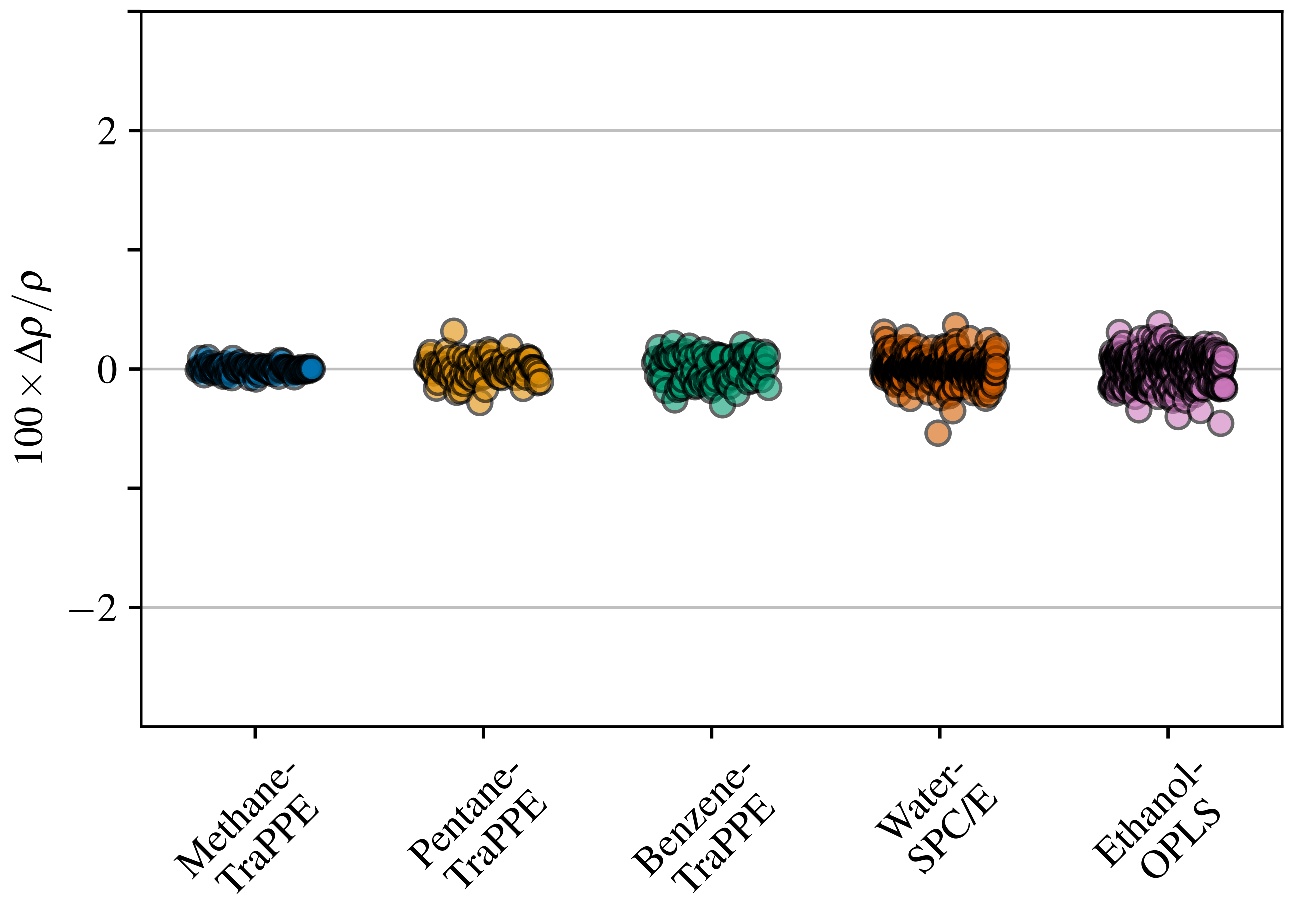

Figure S11: Simulation relative deviation for MoSDeF models-force fields when grouping MC or MD engines separately.

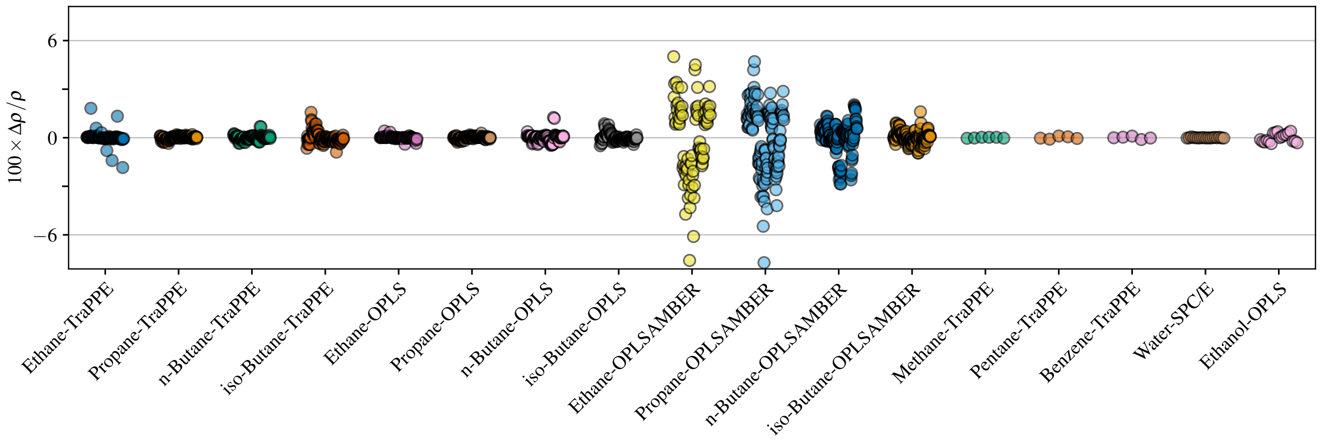

Figure S12: RR molecules-force field relative errors while grouping MC and MD engines together (as opposed to Figure 14).

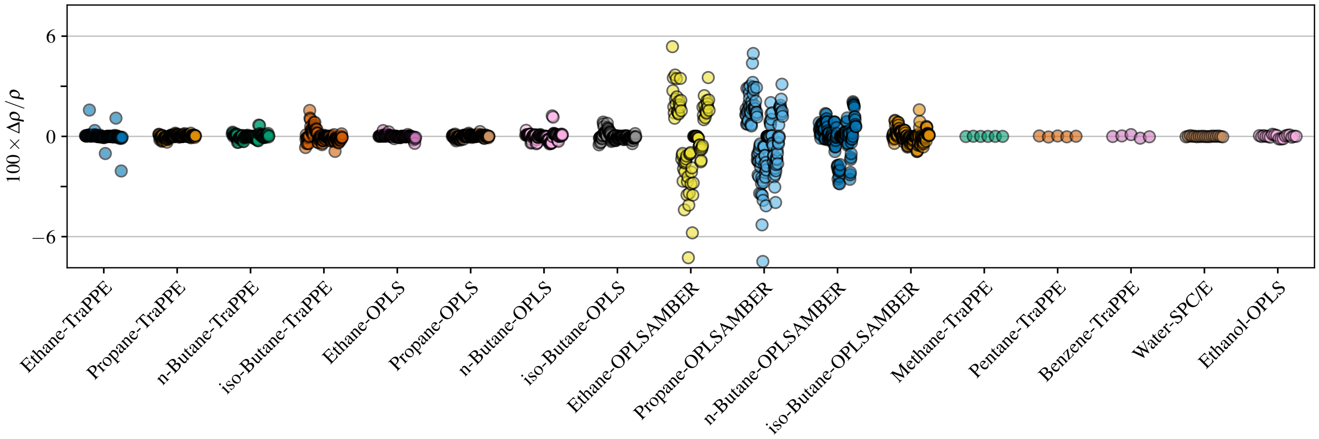

Figure S13: RR molecules-force field relative errors while grouping MC and MD engines separately.

Literature Cited

(1) Merz, P. T.; Shirts, M. R. Testing for physical validity in molecular simulations. *PloS one* **2018**, *13*, e0202764.

(2) Jorgensen, W. L.; Tirado-Rives, J. The OPLS [Optimized Potentials for Liquid Simulations] Potential Functions for Proteins, Energy Minimizations for Crystals of Cyclic Peptides and Crambin. *J. Am. Chem. Soc.* **1988**, *110*, 1657–1666.

(3) Weeks, J. D.; Chandler, D.; Andersen, H. C. Role of Repulsive Forces in Determining the Equilibrium Structure of Simple Liquids. *J. Chem. Phys.* **1971**, *54*, 5237–5247.

(4) Bussi, G.; Donadio, D.; Parrinello, M. Canonical sampling through velocity rescaling. *J. Chem. Phys.* **2007**, *126*, 014101.

(5) Martyna, G. J.; Tobias, D. J.; Klein, M. L. Constant pressure molecular dynamics algorithms. *J. Chem. Phys.* **1994**, *101*, 4177–4189.

(6) Anderson, J. A.; Eric Irrgang, M.; Glotzer, S. C. Scalable Metropolis Monte Carlo for simulation of hard shapes. *Computer Physics Communications* **2016**, *204*, 21–30.
